# An H4K16 histone acetyltransferase mediates decondensation of the X chromosome in *C. elegans* males

**DOI:** 10.1186/s13072-016-0097-x

**Published:** 2016-10-19

**Authors:** Alyssa C. Lau, Kevin P. Zhu, Elizabeth A. Brouhard, Michael B. Davis, Györgyi Csankovszki

**Affiliations:** 1 Department of Molecular, Cellular and Developmental Biology, University of Michigan, 830 N. University Ave., Ann Arbor, MI 48109-1048 USA; 2 Genome Technologies, The Jackson Laboratory for Genomic Medicine, Farmington, CT 06032 USA

**Keywords:** *Caenorhabditis elegans*, Dosage compensation, Gene expression, Epigenetics, Chromosome territories, Chromatin, Histone acetylation

## Abstract

**Background:**

In *C. elegans*, in order to equalize gene expression between the sexes and balance X and autosomal expression, two steps are believed to be required. First, an unknown mechanism is hypothesized to upregulate the X chromosome in both sexes. This mechanism balances the X to autosomal expression in males, but creates X overexpression in hermaphrodites. Therefore, to restore the balance, hermaphrodites downregulate gene expression twofold on both X chromosomes. While many studies have focused on X chromosome downregulation, the mechanism of X upregulation is not known.

**Results:**

To gain more insight into X upregulation, we studied the effects of chromatin condensation and histone acetylation on gene expression levels in male *C. elegans*. We have found that the H4K16 histone acetyltransferase MYS-1/Tip60 mediates dramatic decondensation of the male X chromosome as measured by FISH. However, RNA-seq analysis revealed that MYS-1 contributes only slightly to upregulation of gene expression on the X chromosome. These results suggest that the level of chromosome decondensation does not necessarily correlate with the degree of gene expression change in vivo. Furthermore, the X chromosome is more sensitive to MYS-1-mediated decondensation than the autosomes, despite similar levels of H4K16ac on all chromosomes, as measured by ChIP-seq. H4K16ac levels weakly correlate with gene expression levels on both the X and the autosomes, but highly expressed genes on the X chromosome do not contain exceptionally high levels of H4K16ac.

**Conclusion:**

These results indicate that H4K16ac and chromosome decondensation influence regulation of the male X chromosome; however, they do not fully account for the high levels of gene expression observed on the X chromosomes.

**Electronic supplementary material:**

The online version of this article (doi:10.1186/s13072-016-0097-x) contains supplementary material, which is available to authorized users.

## Background

In many organisms, sex is determined by an XY-based system, where females are homogametic (XX) and males are heterogametic (XY). The resulting difference in sex chromosome number is counteracted by modulating gene expression levels in a process called dosage compensation. According to Ohno’s hypothesis, dosage compensation has to balance both X and autosomal (A) expression levels within one sex as well as gene expression between the sexes [1]. Dosage compensation strategies differ among species. *Drosophila melanogaster* males upregulate their single X chromosome by a factor of two, leading to both an X:A gene expression balance in males and an equalization of X-linked gene expression between the sexes [2, 3]. In other species, different mechanisms balance X-linked gene expression between the sexes. Mammalian females silence one X chromosome by X inactivation [4–6], while hermaphrodite *C. elegans* worms repress both X chromosomes twofold [7–9]. In *C. elegans* hermaphrodites, the dosage compensation complex (DCC) localizes to both X chromosomes to achieve the twofold downregulation of X-linked gene expression. DCC localization leads to X chromosome compaction [10], remodeling of X chromosome topology [11] as well as enrichment of H4K20me1 and the depletion of H4K16ac on the X chromosomes [12, 13]. Nuclear organization, mediated by anchoring heterochromatic regions to the nuclear lamina, also contributes to X chromosome repression [14]. In the absence of additional regulatory mechanisms, these processes will equalize gene expression between the sexes, but they will lead to an imbalance between the X and the autosomes.

Whether X upregulation occurs to balance the X to autosomes in mammals and in *C. elegans* is still being debated. Although Ohno published his hypothesis in 1967 [1], it was not until recently that evidence emerged to support the idea in mammals and *C. elegans*. One type of analysis involves comparing average X-linked gene expression levels to average gene expression levels on autosomes. Microarray analysis in mammalian tissues and *C. elegans* showed that in both sexes X-linked genes are expressed at nearly the same levels as autosomal genes, rather than at half the average autosomal level, supporting the idea of X upregulation in both males and females/hermaphrodites [15–17]. Initial analysis of RNA-seq data did not find evidence for X upregulation [18]. However, more recent studies argued that the upregulation of X-linked genes is supported by RNA-seq data in mammals, *C. elegans* and *Drosophila,* when considering the effects of the skewed gene content and regulation of the X chromosome [19, 20]. Global run-on sequencing analysis (GRO-seq) of active transcription in wild-type *C. elegans* hermaphrodites showed that on average, X-linked genes have engaged RNA polymerase II levels comparable to autosomal genes. This implies that the DCC downregulates transcription on the X chromosomes to the level of the autosomes and not to half the level of autosomes. In hermaphrodites lacking dosage compensation, the X chromosomes have higher levels of engaged RNA Pol II compared to autosomes, suggesting that in the absence of X chromosome downregulation, the X chromosomes are indeed highly expressed [21].

Another method to evaluate X upregulation is comparing expression of genes on the X chromosome to expression of the same gene in a species where the orthologous chromosome is an autosome. Extensive transcriptome comparisons of several avian and mammalian species led to the conclusion that X-linked genes were upregulated in marsupials but not in placental mammals, suggesting that upregulation did occur with the evolution of sex chromosomes but not in all lineages [22]. A similar comparison of nematode species found evidence both for and against X upregulation. When excluding germline-repressed genes, all chromosomes showed equivalent levels of gene expression in males and females/hermaphrodites, supporting the X upregulation hypothesis. However, when comparing the expression of one-to-one orthologs located on the X in one species and the autosome in another, the autosomal ortholog was more highly expressed, arguing against X upregulation [23]. Finally, a recent study compared expression levels of X-linked and autosomal transgenes and demonstrated that the X-linked transgenes were subject to DCC-mediated repression in hermaphrodites; however, X upregulation to balance the X to autosomes did not appear to be a similar chromosome-wide mechanism [24]. Overall, evidence suggests that in the absence of the DCC, on average, X-linked genes in *C. elegans* are expressed at a higher level than autosomal genes. However, if X upregulation indeed occurs, it is likely to operate on a gene-by-gene basis rather than chromosome wide. It is also possible that multiple mechanisms are involved, and various combinations of these mechanisms may be regulating different subsets of genes, as proposed by [24]. Male-specific interactions of X-linked sequences with nuclear pore proteins to achieve upregulation were recently suggested to be such a mechanism [25]. However, a different study was unable to replicate key observations relating to peripheral localization of the male X chromosome [24]. Therefore, the mechanisms that result in high levels of X-linked gene expression in *C. elegans* males remain to be elucidated.

In *Drosophila*, X upregulation is male specific and is achieved by the male-specific lethal (MSL) complex. The MSL complex binds to the male X chromosome, which leads to increased levels of H4K16ac by the MYST family histone acetyltransferase (HAT) MOF, as well as enhanced transcription [26, 27]. H4K16ac decondenses the male X chromosome by weakening nucleosome packing in the chromatin fiber [28]. We recently showed that the X chromosome is significantly decondensed in *C. elegans* males as well. Since X repression in hermaphrodites is accompanied by chromosome condensation [10], we hypothesized that X decondensation may accompany upregulation in *C. elegans* males, as in flies. In this paper, we report that the H4K16ac HAT, MYS-1, is a key factor in regulating the architecture of the *C. elegans* male X chromosome. In contrast to the MYST protein MOF/KAT8/Sas2, the global H4K16 histone acetyltransferase in most organisms [29–32], we find that in *C. elegans,* H4K16ac is mediated by the activity of a different MYST family histone acetyltransferase, the homolog of Tip60, MYS-1. Interestingly, while the lack of MYS-1 activity dramatically changes the compaction of the X chromosomes, these structural changes appear to be partially independent of transcription. In animals depleted of MYS-1, gene expression changes are biased toward the X chromosome, but they are relatively modest. We propose that MYS-1/Tip60-mediated H4K16ac is a key factor in *C. elegans* male X decondensation, but it is only a minor contributor to ensuring high levels of X-linked gene expression.

## Results

### MYS-1 activity mediates X chromosome decondensation in males

**Fig. 1:**
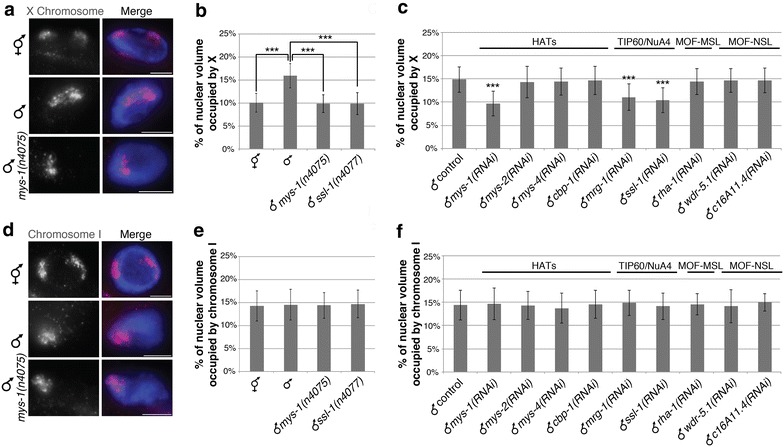
MYS-1 and putative Tip60-/NuA4-like complex members mediate X chromosome decondensation in males. **a**–**c** Adult intestinal nuclei stained with X paint FISH (*red*) to label X chromosome territories and DAPI (*blue*) to label DNA. **a** Representative stained nuclei of wild-type hermaphrodites, wild-type males and *mys*-*1(n4075)* males. *Scale bars* equal 5 µm. **b** Quantification of the percentage of nuclear volume occupied by X in hermaphrodites (number of nuclei (*n*) = 27), males (*n* = 27), *mys*-*1(n4075)* males (*n* = 20) and *ssl*-*1(n4077)* males (*n* = 20). **c** Quantification of the percentage of nuclear volume occupied by X in vector RNAi (*n* = 77), histone acetyltransferases (HATs) and histone acetyltransferases complex member (Tip60/NuA4, MOF-MSL, MOF-NSL) RNAi-treated males. *mys*-*1(RNAi*; *n* = 30), *mys*-*2(RNAi*; *n* = 24), *mys*-*4(RNAi*; *n* = 27), *cbp*-*1(RNAi*; *n* = 26), *mrg*-*1(RNAi*; *n* = 40), *ssl*-*1(RNAi*; *n* = 40), *rha*-*1(RNAi*; *n* = 40), *wrd*-*5.1(RNAi*; *n* = 40), and *c16A11.4(RNAi*; *n* = 40). *Error bars* indicate standard deviation. *Asterisks* indicate level of statistical significance by t-test analysis (***P < .001). **d**–**f** Adult intestinal nuclei stained with chromosome I paint FISH (*red*) to label chromosome I territories and DAPI (*blue*) to label DNA. **d** Representative stained nuclei of wild-type hermaphrodites, wild-type males and *mys*-*1(n4075)* males. *Scale bars* equal 5 µm. **e** Quantification of the percentage of nuclear volume occupied by chromosome I in hermaphrodites (*n* = 20), males (*n* = 17), *mys*-*1(n4075)* males (*n* = 18) and *ssl*-*1(n4077)* males (*n* = 16). **f** Quantification of the percentage of nuclear volume occupied by chromosome I in vector RNAi (*n* = 43), HATs, Tip60/NuA4, MOF-MSL and MOF-NSL member RNAi-treated males. *mys*-*1(RNAi*; *n* = 21), *mys*-*2(RNAi*; *n* = 23), *mys*-*4(RNAi*; *n* = 17), *cbp*-*1(RNAi*; *n* = 28), *mrg*-*1(RNAi*; *n* = 17), *ssl*-*1(RNAi*; *n* = 17), *rha*-*1(RNAi*; *n* = 20), *wrd*-*5.1(RNAi*; *n* = 17), and *c16A11.4(RNAi*; *n* = 20)

We previously reported that the male X chromosome territory is significantly more decondensed in comparison with hermaphrodite Xs [10]. We used a 3D chromosome painting technique, fluorescence in situ hybridization (FISH), to visualize and measure the volumes of chromosomes X and I territories, as described previously [10]. In hermaphrodites, X chromosome DNA takes up 18 % of the genome, yet X chromosome territories were compact and occupied only 10 % of the nuclear volume (number of nuclei (*n*) = 27) due to DCC-mediated compaction. By contrast, in males the single X represents 10 % of the genome, whereas it occupied a significantly larger percentage than the combined percent occupancy of the two X chromosomes in hermaphrodite nuclei (about 16 %, *n* = 27, *P* = 2.41E – 15; Fig. 1a, b, [10, 33]). On the other hand, chromosome I volumes for both hermaphrodites and males were similar and closely correlated with the DNA content of chromosome I (14–16 %; *P* = 0.55; Fig. 1d, e, [10]). These results indicate that the X chromosome in males is more decondensed than in hermaphrodites or what would be predicted based on DNA content.

To search for factors that might lead to decondensation of the male X chromosome, we tested the role of histone acetyltransferases (HATs). Acetylation of histones correlates with chromatin decondensation [34], and H4K16ac is known to be involved in X chromosome upregulation and decondensation in male *Drosophila* [26–28]. Therefore, we performed the same 3D chromosome FISH technique in male worms carrying mutations in or depleted of different histone acetyltransferases (HATs), MYS-1, MYS-2, MYS-4 and CBP-1. MYS-2 is the closest homolog of MOF [35], the fly HAT responsible for H4K16ac on the male X. MYS-1 and MYS-4 are other MYST family histone acetyltransferases [35], and CBP-1 is an unrelated HAT, the homolog of mammalian CBP/p300 [36]. The X chromosome territory in males carrying mutations in, or depleted of MYS-1 by RNAi, occupied a significantly smaller percentage than in control males or males depleted of the other HATs (Fig. 1a–c). In control male worms, X chromosome territories were decondensed with a mean percent nuclear volume of 15.74 ± 2.01 % (*n* = 27), while in *mys*-*1(n4075)* mutant males the X chromosome occupied 9.87 ± 1.97 % (*n* = 20) of the nucleus (*P* = 5.76E − 13), close to the value predicted based on DNA content [10, 33] (Fig. 1a, b). Similar results were seen in male worms depleted of MYS-1, with the X chromosome occupying 9.71 ± 2.68 % (*n* = 30) of the nucleus compared to the 14.85 ± 2.79 % (*n* = 77) in control males fed bacteria carrying an empty vector (*P* = 6.01E − 14). The depletion of other HATs, MYS-2, MYS-4 and CBP-1 showed no significant change in X chromosome territories compared to control males, occupying 14.33 ± 3.39 % (*n* = 24, *P* = 0.45), 14.41 ± 2.93 % (*n* = 27, *P* = 0.49) and 14.64 ± 3.01 % (*n* = 26, *P* = 0.75), respectively (Fig. 1c; Additional file 1: Fig. S1). Additionally, chromosome I remained unaffected in *mys*-*1(n4075)* mutants and in males depleted of MYS-1 (*n* = 21, *P* = 0.75), MYS-2 (*n* = 23, *P* = 0.98), MYS-4 (*n* = 17, *P* = 0.48) and CBP-1 (*n* = 28, *P* = 0.77), with a mean volume of consistently around 14 % in all backgrounds (Fig. 1d–f; Additional file 1: Fig. S1). These results suggest that MYS-1 activity is required for X chromosome decondensation in males and that this activity disproportionately affects the X chromosome compared to autosomes.

### Putative worm Tip60/NuA4 complex members mediate male X chromosome decondensation

MYS-1 is the *C. elegans* homolog of the MYST family HAT Tip60, but also more distantly related to another MYST HAT, MOF, the HAT subunit of MSL and NSL complexes [35]. To determine whether MYS-1 acts in the context of a Tip60-/NuA4-like complex, MOF-MSL-like complex or MOF-NSL-like complex, we examined other putative members of these complexes. We found that only the depletion of Tip60-/NuA4-like complex homologs altered X chromosome territories. Similar to *mys*-*1(RNAi)* males, males depleted of MRG-1 (homolog of MRG15) or SSL-1 (homolog of Domino) showed loss of X chromosome decondensation, with X chromosome territories occupying 11.08 ± 2.86 % (*n* = 40, *P* = 3.28E − 10) and 10.40 ± 2.67 % (*n* = 40, *P* = 2.17E − 13), respectively (Fig. 1c; Additional file 1: Fig. S1). Similarly, in *ssl*-*1(n4077)* mutant males, the X chromosome territory occupied a mean percent nuclear volume of 9.83 ± 2.38 % (*n* = 20, *P* = 6.72E – 12; Fig. 1b). However, when depleting RHA-1 (homolog of MOF-MSL subunit MLE; 14.42 ± 2.83 %, *n* = 40, *P* = 0.42) or WRD-5.1 (homolog of WDS; 14.68 ± 2.59 %, *n* = 40, *P* = 0.75) and C16A11.4 (homolog of MBD-R2; 14.66 ± 2.64 %, *n* = 40, *P* = 0.71), we do not see a loss of decondensation compared to control males (14.85 ± 2.79 %, *n* = 77; Fig. 1c; Additional file 1: Fig. S1). Chromosome I was unaffected in all backgrounds (Fig. 1d–f; Additional file 1: Fig. S1). These results suggest that members of a putative Tip60-/NuA4-like complex may work together to decondense the X chromosome in males.

### MYS-1 influences dosage compensation in hermaphrodites

**Fig. 2:**
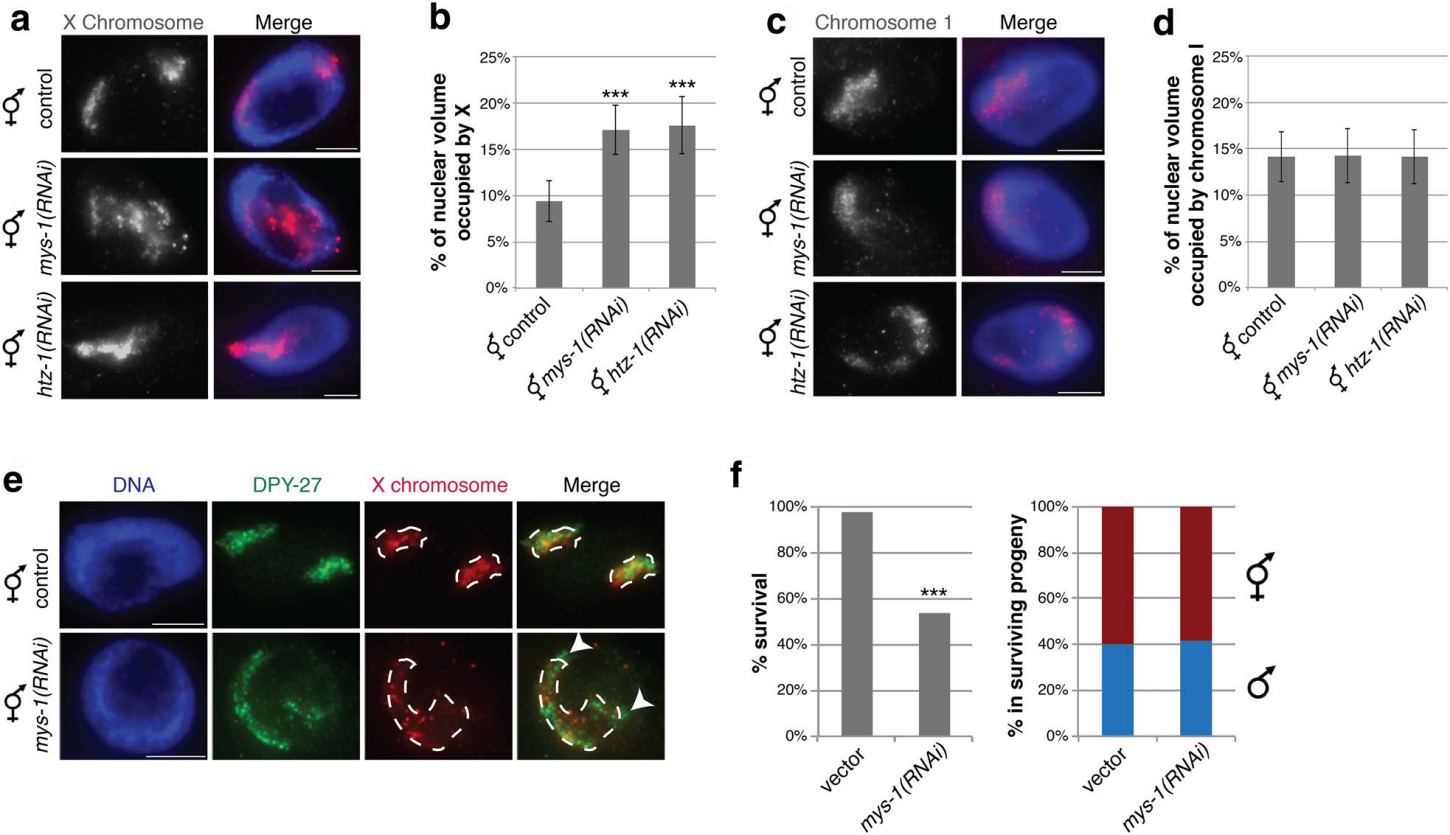
The effects of MYS-1 on hermaphrodite X chromosomes. **a** Representative images of adult hermaphrodite intestinal nuclei in control, *mys*-*1(RNAi)* and *htz*-*1(RNAi)* stained with X paint FISH (*red*) to label X chromosome territories and DAPI (*blue*) to label DNA. *Scale bars* equal 5 µm. **b** Quantification of the percentage of nuclear volume occupied by X in control (*n* = 40), *mys*-*1(RNAi*; *n* = 36) and *htz*-*1(RNAi*; *n* = 37) hermaphrodites. *Error bars* indicate standard deviation. *Asterisks* indicate level of statistical significance by t-test analysis (***P < .001). **c** Representative images of adult hermaphrodite intestinal nuclei in control, *mys*-*1(RNAi)* and *htz*-*1(RNAi)* stained with chromosome I paint FISH (*red*) and DAPI (*blue*) to label DNA. *Scale bars* equal 5 µm. **d** Quantification of the percentage of nuclear volume occupied by chromosome I in control hermaphrodites (*n* = 20), *mys*-*1(RNAi)*(*n* = 20) and *htz*-*1(RNAi*; *n* = 20). *Error bars* indicate standard deviation. No statistically significant differences were found (*P* > 0.5). **e** Immunofluorescence with antibodies against DCC component DPY-27 (*green*), combined with X paint FISH (*red*), and DAPI (*blue*) staining. *White arrowheads* point to regions with strong DCC signal without strong X paint signal. **f** Unmated *him*-*8* hermaphrodites subjected to control vector or *mys-1(RNAi)* were allowed to lay eggs. The percent of progeny that survived to adulthood is shown on the *left*, and the proportion of male and hermaphrodite worms among the surviving progeny is shown on the *right*. *Asterisks* indicate level of statistical significance by Chi-square test analysis (***P < .001)

According to Ohno's hypothesis, X upregulation occurs in both sexes in *C. elegans*. In hermaphrodites, DCC-mediated repression counters this upregulation. To determine how loss of *mys*-*1* activity affects the hermaphrodite X chromosomes, we depleted MYS-1 by RNAi and examined X chromosome structure. Surprisingly, we found that the X in MYS-1-depleted hermaphrodites occupied a significantly larger percentage of the nucleus (17.11 ± 2.66 %, *n* = 36) compared to controls (9.41 ± 2.21 %, *n* = 40, *P* = 4.33E – 22; Fig. 2a, b). By contrast, chromosome I volumes were unchanged at an average of 14 % in all backgrounds (Fig. 2c, d). Since the X volume in MYS-1-depleted hermaphrodites closely parallels the predicted volume based on DNA content (18 %), we hypothesized that DCC-mediated chromosome condensation might be compromised. In fact, we observed that DCC localization to the X chromosome was disrupted in this background. Immunofluorescence using antibodies specific to DPY-27, a DCC component, combined with X paint FISH revealed that in *mys*-*1*(*RNAi*), the DCC localization is not strictly limited to the X chromosomes as it is in controls (Fig. 2e). This phenotype is similar to what we previously described for worms carrying mutations in the histone variant *H2A.Z/htz*-*1* [37]. Combined, these observations suggest that disrupting the chromatin environment of the X chromosomes may contribute to regulating DCC localization. Consistent with the hypothesis that HTZ-1 and MYS-1 affect DCC functions to similar degrees, X chromosome volumes in HTZ-1-depleted hermaphrodites (17.57 ± 3.08 %, *n* = 37) were comparable to the MYS-1 depletions. Note that this value correlates with DNA content, therefore the X chromosomes in these backgrounds are neither condensed nor decondensed, compared to genomic average. Interestingly, X chromosome volumes in *mys*-*1* or *htz*-*1* RNAi conditions are similar to what we previously reported in DCC mutants or depletions [10]. It is possible that our assay is not sensitive enough to distinguish between various degrees of compaction.

The Tip60 complex in other organisms plays many roles, including cell cycle regulation and DNA repair [38]. Consistent with that, *mys*-*1* and *ssl*-*1* mutant males and hermaphrodites are sickly in appearance, and while maternal contribution of wild-type gene product allows the worms to survive to late larval/young adult stage, homozygous mutant hermaphrodites do not produce viable progeny. To examine whether males are more severely affected by the lack of MYS-1 activity, we depleted MYS-1 in a *him*-*8* mutant strain. HIM-8 functions during meiosis, and in its absence, X chromosome segregation is impaired, causing unmated hermaphrodites to produce about 40 % XO male progeny, compared to <1 % in wild type [39]. Consistent with an essential function in both sexes, MYS-1 depletion in this strain led to significant embryonic and larval lethality, as only 54 % (*n* = 1071) of the progeny survived to adulthood, compared to 98 % survival in control vector depletion (*n* = 2090; Fig. 2f, *P* < 0.001, Chi-square test). However, among the surviving progeny, a comparable percentage was male in both conditions (40 % in control, 42 % in *mys*-*1(RNAi)*, not significant, Chi-square test), indicating that the sex-non-specific roles of MYS-1 are more essential to survival than any sex-specific functions that may exist (Fig. 2f). Due to the additional roles of MYS-1 in regulating DCC localization in hermaphrodites described above, we concentrated on analysis of the male X chromosome for the rest of this study.

### Distance measurements confirm Tip60-mediated decondensation of the male X

**Fig. 3:**
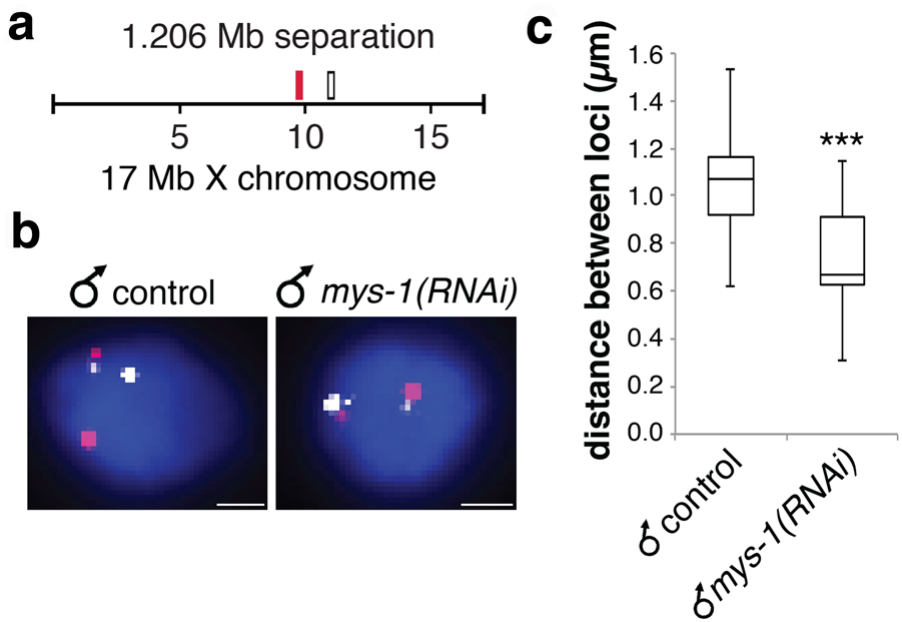
Male X chromatin decondensation is evident at the genomic scale of 1.2 Mb. **a** FISH probe pairs across the X chromosome. The position of YAC probes (*red* and *white boxes*) used in FISH is indicated. **b** 2D projections of 3D stacked images. Representative tetraploid nuclei of adult males fed vector RNAi and *mys*-*1(RNAi)* stained with probe pairs across the X chromosome (*red* and *white*) and counterstained with DAPI (*blue*) to label DNA. *Scale bars* equal 1 µm. **c** Boxplots indicating the distribution of 3D loci distances of male vector RNAi (*n* = 20) and *mys*-*1(RNAi*; *n* = 16) diploid nuclei. *Boxes* show the median and interquartile range of the data. *Asterisks* indicate level of statistical significance by t-test analysis (***P < .001)

To confirm the X decondensation phenotype using a different assay, we performed 3D FISH with pairs of X chromosome YAC probes separated by a genomic distance of 1.2 Mb (Fig. 3a). Previously we found that this genomic distance had the most significant differences between control and DCC mutant hermaphrodites [10]. We analyzed this pair of probes in control male and *mys*-*1(RNAi)* male hypodermal hyp 7 nuclei. These hypodermal cells are tetraploid; therefore, we expected to see two spots for each X-linked probe in male nuclei [40]. We detected a significant decrease in loci distance in *mys*-*1(RNAi)* males compared to control males, with the median distance of 1.06 µm (*n* = 20) between loci in the control males and a median distance of 0.67 µm (*n* = 16) in *mys*-*1(RNAi)* males (*P* = 1.51E – 4; Fig. 2b, c). The closer proximity of the two loci found in *mys*-*1(RNAi)* males correlates with the compact X chromosomes found in the males mutated or depleted of the putative worm Tip60/NuA4 complex members.

### MYS-1 acetylates H4K16

Tip60 is known to acetylate H2AK5 and H4K5, K8, K12 and K16 in vitro [41–43]. To determine what histone marks depend on MYS-1 activity in vivo in *C. elegans*, we performed a combination of immunofluorescence microscopy (IF) and western blot analysis with antibodies specific for different histone marks in wild-type and *mys*-*1(RNAi)* or *mys*-*1(n4075)* mutant worms (Additional file 2: Fig. S2). First, we tested H4K16ac because of its known role in X upregulation and decondensation in flies [26–28]. Indeed, mutations or depletion of MYS-1 led to greatly reduced levels of H4K16ac by IF and western blot analysis, whereas other acetylation marks on H2A, H3 or H4 showed no reduction. We also found that mutations in *mys*-*2*, the closest homolog of the *Drosophila* H4K16ac HAT MOF, led to no reduction in H4K16ac compared to wild type (Additional file 2: Fig. S2). These results suggest that MYS-1 is the major H4K16 HAT in *C. elegans*. Interestingly, H3K14ac and H3K56ac levels increase in MYS-1 mutants compared to wild type, perhaps as part of a compensatory mechanism for the loss of H4K16ac. Overall, these results suggest that similar to *Drosophila*, H4K16ac may be a key mediator in *C. elegans* X chromosome decondensation.

### The X chromosome of XO hermaphrodites phenocopies the male X

To test whether it is the male (XO) karyotype or the male physiology that drives X decondensation, we examined a mutant strain, *her*-*1(e1520)*; *sdc*-*2(y74)*. This strain is karyotypically male (XO), but is transformed into a hermaphrodite by a genetic mutation in the male sex determination pathway [44]. Similar to males, MYS-1-mediated X chromosome decondensation is evident in XO hermaphrodites. X chromosome territories were decondensed in *her*-*1(e1520)*; *sdc*-*2(y74)* mutants occupying a mean percent of 15.42 ± 2.49 % (*n* = 20), while X chromosome decondensation was lost in *her*-*1(e1520)*; *sdc*-*2(y74)* mutants depleted of MYS-1, with X chromosome territories occupying a mean percentage of 9.43 ± 3.02 % (*n* = 20, *P* = 3.89E − 8). This change in chromatin volumes was X specific, with chromosome I occupying a mean percent of 14.65 ± 2.42 % (*n* = 20) and 14.11 ± 2.57 % (*n* = 16, *P* = 0.53), in *her*-*1(e1520)*; *sdc*-*2(y74)* mutants and *her*-*1(e1520)*; *sdc*-*2(y74)* mutants depleted of MYS-1, respectively (Additional file 3: Fig. S3). We demonstrate that *her*-*1(e1520)*; *sdc*-*2(y74)* hermaphrodites (hereinafter referred to as XO hermaphrodites) have the same X chromosome phenotypes as males. It is therefore the male karyotype, not male physiology, that drives X decondensation. Since it is not possible to grow a large pure population of males, but we can grow large pure populations of XO hermaphrodites, we used XO hermaphrodites in many subsequent experiments.

### X compaction and decondensation are initiated at the same time in development

**Fig. 4:**
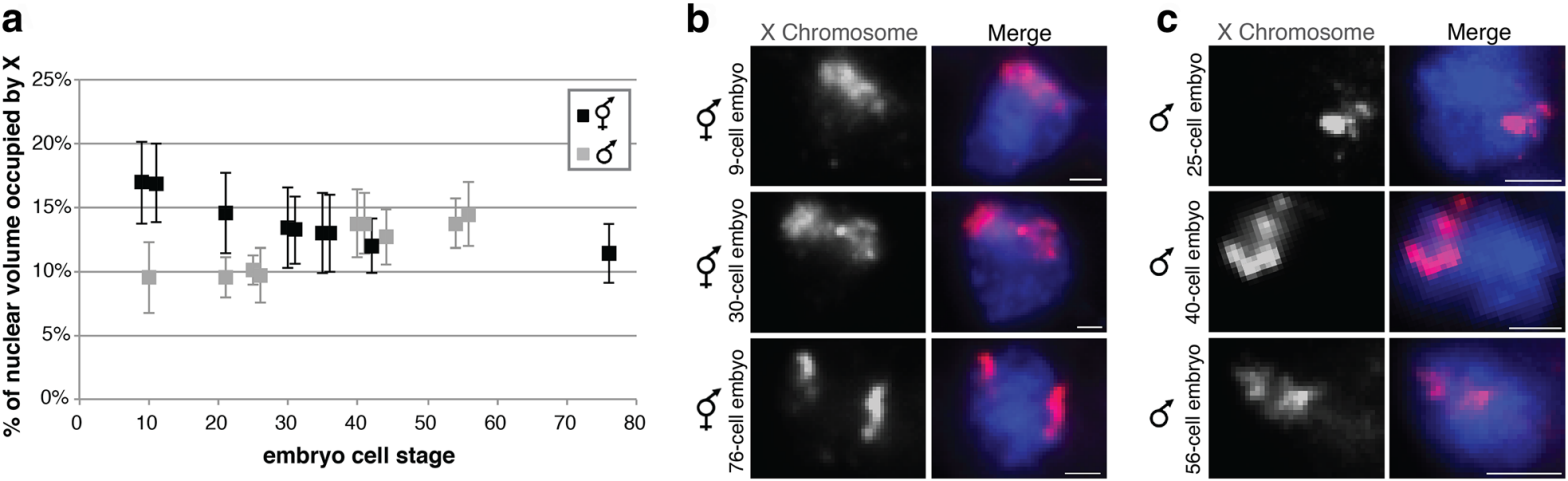
Hermaphrodite X chromosome compaction and male X chromosome decondensation occur simultaneously in development. Hermaphrodite and male embryos stained X paint FISH (*red*) to label X chromosome territories and DAPI (*blue*) to label DNA. **a** Plot of quantified percentages of nuclear volume occupied by X in wild-type hermaphrodite embryos (*black*) and male embryos (*gray*). *Each point* represents one embryo (*n* = 10 nuclei per embryo). *Error bars* indicate standard deviation. **b** Representative stained nuclei of wild-type hermaphrodites at various developmental stages (9 cell, 30 cell, 76 cell). *Scale bars* equal 5 µm. **c** Representative stained nuclei of male *him*-*8(e1489)* animals at various developmental stages (25 cell, 40 cell, 56 cell). *Scale bars* equal 1 µm

In order to determine the developmental timing of DCC-mediated X compaction in hermaphrodites and MYS-1-mediated X decondensation in males, we examined the X chromosomes in hermaphrodite and male embryos at different developmental stages. We found that in both hermaphrodite and male early-stage embryos, the X chromosome volume was similar to what is expected based on DNA content (about 10 % males and 18 % in hermaphrodites), indicating uniform levels of genome compaction in early-stage embryos. In males, we found that the onset of X decondensation occurs rather abruptly, between the 25- and 40-cell stages (Fig. 4a, c). Nuclei in a 25-cell male embryo have a compact X chromosome occupying 10.10 ± 1.17 %, while nuclei in a 40-cell or 56-cell embryo have a decondensed X chromosome occupying 13.74 ± 2.69 and 14.49 ± 2.52 %, respectively (*n* = 10 nuclei at each stage). The fact that X decondensation does not initiate until the 25- to 40-cell stage, may explain why X decondensation was not seen in male embryos in a previous study [25]. X chromosomes in hermaphrodites compact gradually between the 20- and 75-cell stages (Fig. 4a, b). Nuclei in a 9-cell embryo have X chromosomes occupying 16.95 ± 3.22 %, indicating compaction level at genomic average, whereas nuclei in a 30-cell embryo have more condensed X chromosomes occupying 13.45 ± 3.16 %. At the 76-cell stage, nuclei have compact X chromosomes occupying 11.42 ± 2.34 % (*n* = 10 nuclei at each stage), similar to the X chromosome volume in adult wild-type hermaphrodite intestinal nuclei or tail-tip hypodermal cells [10]. The timing of X condensation coincides with DCC localization to the X in hermaphrodites, which begins at the 30- to 50-cell stage [45, 46], and the stage when DCC-mediated repression becomes measurable by RNA-seq analysis [47]. Thus, the X chromosomes in hermaphrodites do not follow a two-step process of decondensation followed by condensation, as the “upregulation followed by downregulation” model may suggest. Rather X compaction (in hermaphrodites) and X decondensation (in males) occur around the same time in development. If MYS-1 activity promotes decondensation of the X in hermaphrodites, it is likely counteracted by simultaneously occurring DCC-mediated compaction.

### Transcription is not required for the initiation or maintenance of X decondensation

**Fig. 5:**
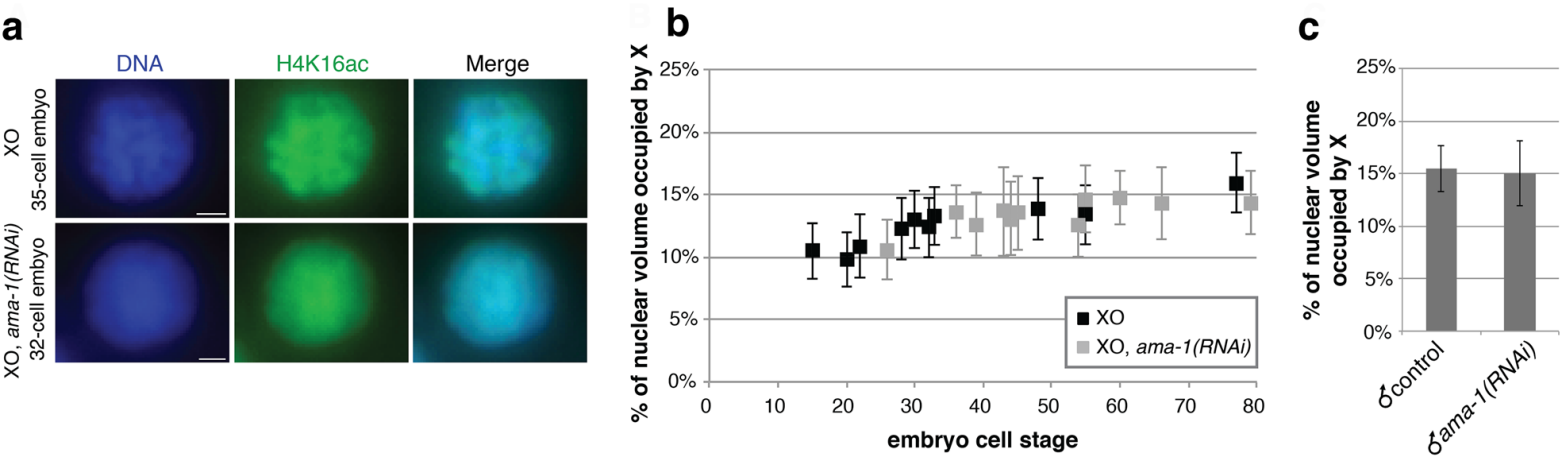
Transcription is not required for the initiation or maintenance of X decondensation. **a** H4K16ac immunofluorescence stained nuclei of XO embryos and XO embryos fed *ama*-*1(RNAi)*. H4K16ac levels did not decrease in XO embryos fed *ama*-*1(RNAi)*. **b** Plot of quantified percentages of nuclear volume occupied by X in XO embryos fed vector RNAi (*black*) and XO embryos fed *ama*-*1(RNAi*; *gray*). *Each point* represents one embryo (*n* = 10 nuclei per embryo). *Error bars* indicate standard deviation. **c** Quantification of the percentage of nuclear volume occupied by adult males fed vector RNAi (*n* = 20) and *ama*-*1(RNAi*; large subunit of RNA polymerase II; *n* = 20). *Error bars* indicate standard deviation

The onset of X decondensation in males is in the same time window when zygotic transcription of a significant number of X-linked genes is initiated [48] (Fig. 4), which suggests that transcription may drive decondensation. Indeed, in mammals, inhibition of transcription leads to increased compaction chromosome wide both for an autosome [49] and for the active X chromosome [50]. To test this hypothesis, we examined XO hermaphrodite embryos depleted of AMA-1, the large subunit in RNA polymerase II. Lack of zygotic transcription due to AMA-1 depletion causes embryonic lethality by 100-cell stage [51]. Our AMA-1 depletion was effective, as high embryonic lethality was evident. However, H4K16ac levels did not decrease (Fig. 5a). The onset of X chromosome decondensation occurs between the 25- and 40-cell stages in XO hermaphrodite embryos (Fig. 5b), similar to that was observed in males (Fig. 4). Furthermore, when transcription is severely reduced in XO hermaphrodites depleted of AMA-1, X decondensation still occurs between the 25- and 40-cell stage (Fig. 5b). These results suggest that normal levels of transcription are not required for H4K16ac and for the initiation of X decondensation; therefore, decondensation is not simply a consequence of high levels of transcriptional activity.

To test whether continued transcription is needed to maintain decondensed X chromosome territories in adult worms, we fed L1 stage worms *ama-1(RNAi)* and empty vector and analyzed these worms once they reached young adulthood. AMA-1 depletion was effective, as the fed hermaphrodites laid dead embryos. The X chromosome territories in intestinal cells of adult males depleted of AMA-1 showed no significant difference compared to the X chromosome territories in control males (Fig. 5c). The X chromosome territory occupied a mean percent nuclear volume of 15.45 ± 2.20 % (*n* = 20) in control males and 15.05 ± 3.11 % (*n* = 20, *P* = 0.63) in *ama*-*1(RNAi)* males. This finding suggests that continued high levels of transcription are not required for the maintenance of male X chromosome decondensation.

### H4K16ac is uniformly distributed in the nucleus

**Fig. 6:**
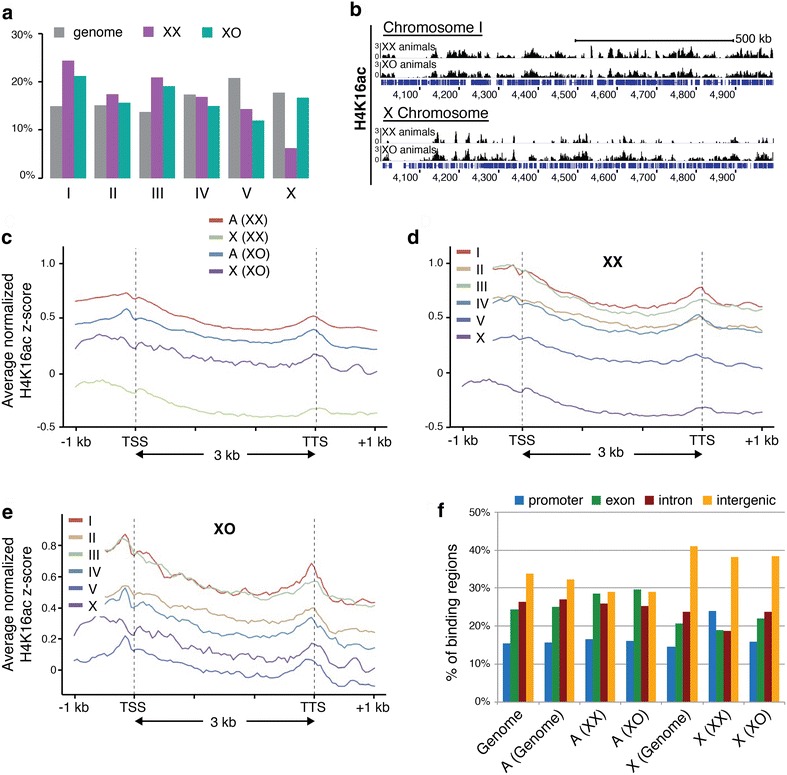
Profile of H4K16ac in XX and XO worms. H4K16ac ChIP-seq in XX and XO hermaphrodites. **a** Chromosomal distributions of H4K16ac ChIP regions compared to the fraction of the genome found on each chromosome. **b** Representative IGV genome browser views of ChIP-seq scores for H4K16ac. **c** Average normalized H4K16ac ChIP-seq enrichment scores plotted for X and autosomes across the gene body. **d** Average normalized H4K16ac ChIP-seq enrichment scores plotted for each chromosome across the gene body of expressed genes (RPKM > 1) in XX hermaphrodites and **e** XO hermaphrodites. **f** Percentage of H4K16ac peak found across each category of genomic sequence on the X and autosomes in XX and XO hermaphrodites compared to the fraction of the total genome and the X and A genome fraction found in each genomic category

To examine whether the greater sensitivity of the male X (compared to autosomes) to the loss of H4K16ac is reflected in higher levels of H4K16ac on the chromosome, we investigated the distribution of this mark genome wide. We have previously shown by immunofluorescence that H4K16ac levels are depleted on the X chromosome in wild-type XX animals due to dosage compensation, but not in XO males [13]. For higher-resolution analysis, we performed ChIP-seq of H4K16ac in wild-type XX hermaphrodites and XO hermaphrodites collected at the L3 larval stage. Using single-end sequencing we could uniquely align 78–92 % of the 18–28 million reads to the genome for three replicates in each background. After normalization, the ratio of the fraction of reads that mapped to the X chromosome divided by the X chromosome genome fraction was 0.33 in XX hermaphrodites and 1.01 in XO hermaphrodites, mirroring previous immunofluorescence data [13]. We examined the peak distribution of H4K16ac by plotting the chromosomal distributions of H4K16ac ChIP regions (Fig. 6a). Consistent with what was previously seen by immunofluorescence, there were fewer H4K16ac peaks on the X (approximately 52 peaks/Mb) compared to the autosomes (approximately 95 peaks/Mb), and the fraction of H4K16ac peaks on the X are significantly less than the fraction of the genome found on the X chromosome in XX hermaphrodites (Fig. 6a). In XO hermaphrodites, the distribution of H4K16ac on the X chromosome is almost equivalent to the genome fraction, consistent with lack of DCC-mediated reduction of H4K16ac in this background. However, H4K16ac does not appear to be more highly distributed on the X chromosome compared to the autosomes in XO hermaphrodites. Representative genome browser views of ChIP-seq scores for H4K16ac were used to visualize signal intensities of H4K16ac peaks (Fig. 6b). H4K16ac binding across the X chromosome is underrepresented compared to autosomes in XX animals, whereas X and autosomal signal intensities are similar in XO hermaphrodites. Additionally, H4K16ac binding sites and intensities are similar across the autosomes in both XX and XO hermaphrodites. On the X chromosome, H4K16ac is enriched in XO hermaphrodites compared to XX hermaphrodites. These results suggest that despite greater sensitivity to MYS-1 activity in terms of chromosome condensation, the X chromosome of XO animals overall does not have higher levels of H4K16ac than autosomes.

Next, we examined H4K16ac levels around the annotated genes to determine whether differences can instead be observed in transcribed regions. It has been previously reported in mouse embryonic stem (ES) cells and human CD4+ T cells that H4K16ac peaks are seen around the transcription start site (TSS) [52–54]. In flies, H4K16ac is found at the TSS and accumulates toward the 3′ end of coding regions on the single male X, whereas on autosomes and in females H4K16ac is detected only at the promoters [55–58]. To examine the global profile of H4K16ac on transcribed regions, we created normalized z-scores for regions across gene bodies and extending 1 kb upstream of the TSS and 1 kb downstream of the transcription termination site (TTS). Metagene profiles derived from an average of all expressed genes in the genome showed that H4K16ac levels on X and autosomal-linked genes peak near the TSS in both XX and XO hermaphrodites. In XX animals, H4K16ac levels around transcribed regions are depleted on X-linked genes compared to autosomal genes, whereas in XO hermaphrodites, H4K16ac levels on the X chromosome are almost equivalent to the average autosomal levels around transcribed regions (Fig. 6c). When examining chromosomes separately, X-linked genes on average have lower levels of H4K16ac compared to all autosomes in XX animals (Fig. 6d). In XO hermaphrodites, H4K16ac levels are enriched on the X compared to chromosome V and more comparable to levels on other autosomes (Fig. 6e). These results suggest that H4K16ac is not enriched on X-linked genes compared to the autosomal genes in XO hermaphrodites, and differences in H4K16ac levels in genic regions are not the cause of the observed X-specific decondensation.

To obtain an overview of the fraction of X and autosomal binding across the genomic regions, we classified H4K16ac binding regions into four categories: promoter, exon, intron and intergenic. Relative to autosomes, the X chromosome contains proportionally more intergenic regions and has a correspondingly higher fraction of H4K16ac peaks located in the intergenic regions in both XX animals and XO hermaphrodites (Fig. 6f). These results indicate that although intergenic regions are not more highly acetylated on the X than on autosomes, the X chromosomes have more acetylatable intergenic regions that may contribute to regulating higher-order chromosome structure. We do not currently know which intergenic sites might influence chromatin architecture, but one candidate is boundary elements between topologically associating domains (TADs). In XX hermaphrodites, the DCC initially binds to both X chromosomes via sequence-specific recruitment to *rex* sites. These sites are often found in intergenic regions, and many coincide with boundaries between TADs. Long-range interactions between *rex* sites mediated by the DCC are proposed to reshape X chromatin structure in wild-type hermaphrodites [11]. This led us to investigate H4K16ac binding surrounding *rex* sites in XX and XO hermaphrodites. We examined *rex* sites at TAD boundaries that have the strongest *rex*–*rex* interactions as categorized by Crane et al. [11]. Genome browser views of ChIP-seq scores for H4K16ac revealed that in XX hermaphrodites, H4K16ac is absent in the region surrounding *rex* sites, while in XO hermaphrodites, H4K16ac is often but not always present near *rex* sites (Additional file 4: Fig. S4). The differences in acetylation at these sites could potentially explain the difference in X chromosome condensation in XX and XO animals [10]. The differences in acetylation at yet undefined intergenic sites may explain the differences in compaction between the X and the autosomes in XO animals.

### MYS-1 activity is biased toward upregulation of X-linked genes

**Fig. 7:**
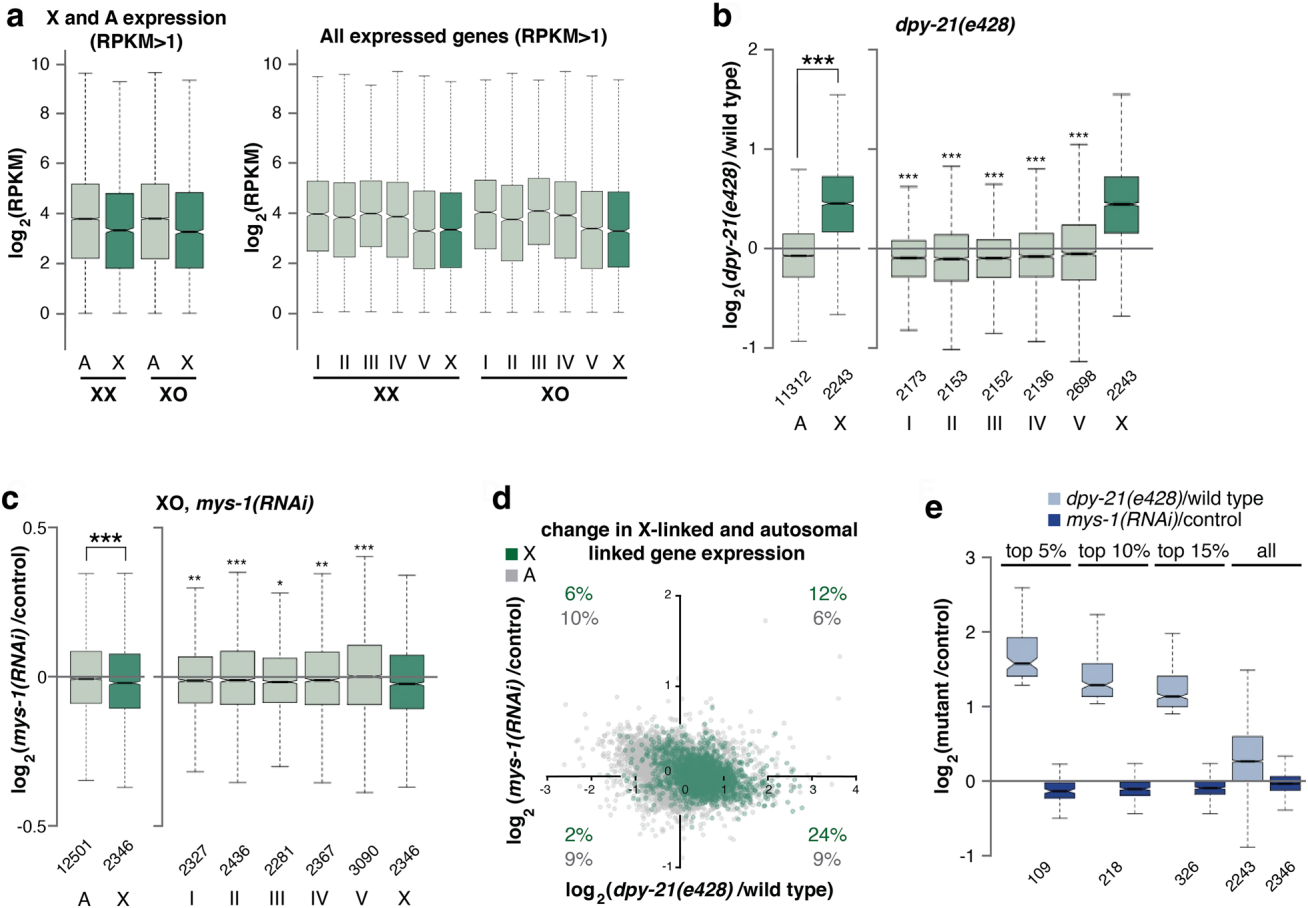
RNA-seq analysis of gene expression changes in MYS-1-depleted XO worms. **a** X and autosome expression levels of genes with RPKM > 1 and expressed genes on individual chromosomes in XX and XO hermaphrodites. **b** Boxplot shows the distribution of log_2_ expression ratios on the X, autosomes and all separate chromosomes between *dpy*-*21(e428)* mutant XX hermaphrodites and wild-type XX hermaphrodites. X chromosome was significantly derepressed compared to the autosomal average and all other chromosomes. Increased expression from the X was tested between X and autosome by one-sided Wilcoxon rank-sum test (***P < .001). **c** Boxplot shows the distribution of log_2_ expression ratios on the X, autosomes and all separate chromosomes between XO hermaphrodites fed *mys*-*1(RNAi)* and XO hermaphrodites fed vector RNAi. X chromosome was statistically significantly repressed compared to the autosomal average and all other chromosomes, although the degree of repression was minor. Decreased expression from the X was tested between X and autosomes by one-sided Wilcoxon rank-sum test (*P < .05; **P < .01; ***P < .001). **d** The magnitude of log_2_ expression ratios of X-linked (*green*) and autosomal genes (*gray*) between XO hermaphrodites fed *mys*-*1(RNAi)* and control XO plotted against *dpy*-*21(e428)* mutants and wild-type animals. The percentages of X-linked and autosomal genes with >10 % change in expression (±0.1 in log_2_) in both knockdown and mutant compared to control worms are indicated in each quadrant. **e** Boxplots show the distribution of log_2_ ratios on the X between *dpy*-*21(e428)* mutants and wild-type animals and *mys*-*1(RNAi)* XO hermaphrodites and control XO hermaphrodites. The first 3 sets of boxplots show the distribution of log_2_ ratios of the top 5 %, top 10 % and top 15 % of highly differentially expressed X-linked genes in *dpy*-*21(e428)* mutants. The last set shows the distribution of all X-linked genes

To analyze how gene expression levels are regulated on the X chromosome, we first performed mRNA-seq analysis and compared average gene expression levels of all X and autosomal-linked genes in XX and XO hermaphrodites. In *C. elegans*, it is difficult to compare X and autosomal expression levels in whole adult worms due to X chromosome silencing in germ cells by a process unrelated to dosage compensation [59]. Germline proliferation begins at the L1 larval stage and becomes more rapid after the L3 stage [60]. By the L4 stage, there are about equal number of germ and somatic cells in the worm. At the L4, but not at the L3 stage, the presence of the germline greatly affects measured X:A expression values [19]. We therefore analyzed gene expression in L3 worms, which have approximately 60 germ cells and 700 somatic cells [61]. Therefore, RNA levels at this stage should be dominated by somatic gene expression. Consistent with previous data, we observed a median X:A expression ratio of 0.88 in wild-type XX animals and 0.86 in XO hermaphrodites (Fig. 7a) [18, 19]. These values are significantly higher than 0.5, which would be expected if the X chromosomes are expressed at the same level as autosomes in XO hermaphrodites and are subjected to DCC-mediated repression in XX hermaphrodites. Examining each chromosome individually revealed variations among average gene expression levels on autosomes, but again the X was more highly expressed than half the autosomal level. These results are consistent with previous observations [15, 19, 23] that X-linked genes, on average, are more highly expressed than autosomal genes in XO animals.

In order to study effects of MYS-1 on X chromosome expression, we analyzed gene expression changes in L3 stage MYS-1-depleted XO hermaphrodites by RNA-seq analysis. X-linked genes that are upregulated in XO animals should be the same genes that are repressed by the DCC in XX animals. Therefore, in order to compare the genes affected by MYS-1 in XO worms to the genes affected by DCC-mediated repression in XX animals, we also analyzed *dpy*-*21(e428)* mutant XX hermaphrodites. DPY-21 is a non-condensin protein member of the DCC. These null mutants (*e428*) are viable; however, dosage compensation function is disrupted [62]. At the L3 stage, *dpy*-*21(e428)* mutation caused X chromosome derepression, and we observed an increase in average X-linked gene expression compared to gene expression changes on autosomes. The median log_2_ ratio of expression between *dpy*-*21(e428)* worms and wild-type XX hermaphrodites was significantly higher on the X (0.447) compared to autosomes (−0.080) and compared to each chromosome (Fig. 7b, one-sided Wilcoxon rank-sum test *P* < .001). These results are similar to previously reported data of dosage compensation-mediated gene expression changes [11, 47, 63]. To test how MYS-1 effects gene expression in XO hermaphrodites, we depleted MYS-1 by RNAi. Our RNAi conditions disrupted *mys*-*1* function sufficiently, such that protein levels decreased to below the level of detection by western blot analysis (Additional file 5: Fig. S5). Contrary to the X chromosome derepression found in *dpy*-*21(e428)* hermaphrodites, but consistent with role for MYS-1 in X upregulation, depleting MYS-1 in XO hermaphrodites caused a small decrease in the average expression levels of X-linked genes. We detected a very small decrease in average X-linked gene expression relative to gene expression changes on autosomes. The median log_2_ ratio of expression between *mys*-*1(RNAi)* XO hermaphrodites and control XO hermaphrodites was significantly lower on the X (−0.021) compared to autosomes (−0.008) and compared to each chromosome (Fig. 7c, one-sided Wilcoxon rank-sum test *P* < .05). Thus, MYS-1 contributes to the observed higher level of gene expression on the X chromosome in XO hermaphrodites, but the effects are minor. It should be noted, however, that despite a significant decrease in protein levels (Additional file 5: Fig. S5), RNAi depletion is an incomplete knockdown of gene function. It remains possible that the effects on gene expression would be more substantial in null mutants.

Next, we set out to examine whether MYS-1 contributes to upregulation of the same set of genes as are repressed by the DCC. We plotted the log_2_ ratio of expression of *mys*-*1(RNAi)* XO hermaphrodites and control XO hermaphrodites against the log_2_ ratio of expression of *dpy*-*21(e428)* mutants and wild-type XX hermaphrodites. A larger subset of X-linked genes (24 vs. 2–12 % for genes in other quadrants) were both derepressed in *dpy*-*21(e428)* mutants and had reduced expression in *mys*-*1(RNAi)* XO hermaphrodites, whereas autosomal genes did not show such an effect (Fig. 7d). Additionally, the X-linked genes that are most affected by the DCC in hermaphrodites are also more highly upregulated in XO hermaphrodites by MYS-1. The top 5 %, top 10 % and top 15 % of highly differentially expressed X-linked genes identified in *dpy*-*21(e428)* mutants are also more highly differentially expressed in *mys*-*1(RNAi)* XO hermaphrodites (Fig. 7e). We conclude that MYS-1 activity causes a mild upregulation of X-linked genes and that the genes most affected by the loss of dosage compensation in *dpy*-*21(e428)* mutants are also the genes whose expression is most upregulated by MYS-1 in XO hermaphrodites. However, MYS-1 activity alone does not explain the significantly higher level of gene expression on the X chromosome of XO animals. Thus, the X chromosome-specific decondensation mediated by MYS-1 is only accompanied by a small degree of bias toward regulating X-linked genes.

### Similar correlations of H4K16ac and gene expression on the X and autosomes

**Fig. 8:**
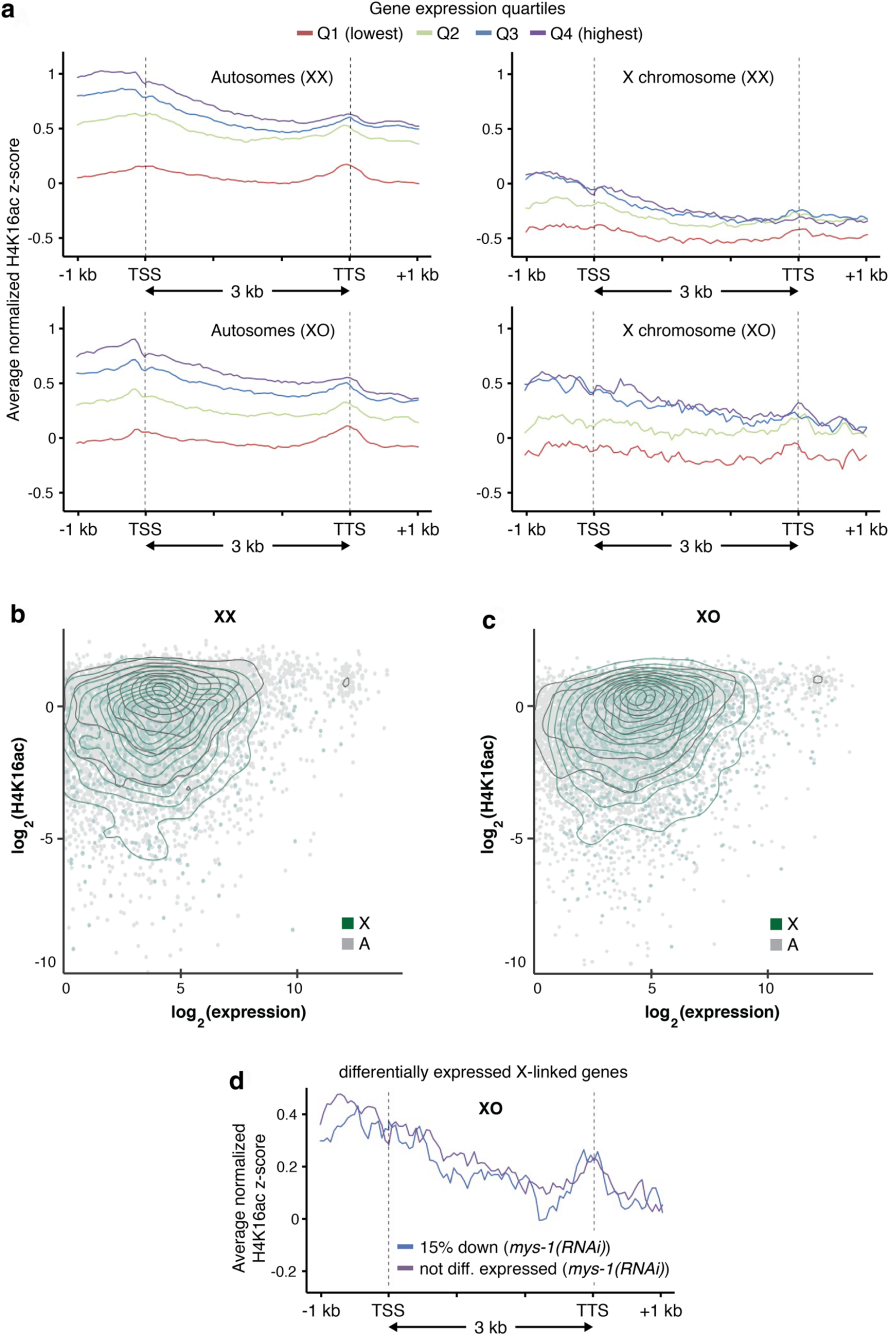
Relationships between levels of H4K16ac and gene expression. **a** Average normalized H4K16ac ChIP-seq enrichment scores separated into quartiles according to expression plotted for X and autosomes in XX and XO hermaphrodites. **b** Average H4K16ac ChIP score within 500 bp upstream of the TSS plotted against the RNA level of each gene for XX and **c** XO hermaphrodites. Expressed autosomal genes (RPKM > 1) are represented as *gray points*, with point density shown by *black line* contour. Expressed X-linked (RPKM > 1) are represented by *green dots* and *green line contour*. **d** Average normalized H4K16ac ChIP-seq enrichment scores in XO hermaphrodites separated by the top 15 % downregulated X-linked genes in *mys*-*1(RNAi)* XO hermaphrodites and X-linked genes that were not differentially expressed

We next wanted to compare the relationship between H4K16ac binding and gene expression on the X to the relationship on autosomes. We generated H4K16ac profiles for gene bodies for X and autosomal-linked genes subdivided by quartiles of expression. In both XX and XO hermaphrodites, regardless of whether they were X-linked genes or autosomal genes, H4K16ac positively correlated with gene expression (Fig. 8a). These results are consistent with the patterns previously reported in mouse ES cells and human CD4+ T cells and with the involvement of H4K16ac in transcriptional activation [52–54]. We note, however, that the top two quartiles had essentially identical levels of H4K16ac on the X chromosome, suggesting that the most highly expressed genes on the X do not have exceptionally high levels of H4K16ac. We then plotted the average ChIP score within 500 bp upstream of the TSS against the RNA levels of X and autosomal genes for pairwise comparisons between H4K16ac binding and gene expression (Fig. 8b, c). There were slight dependencies between H4K16ac and gene expression, consistent with an overall correlation between the two. However, overall X and autosomal genes have similar relationships between H4K16ac and transcription.

Lastly, to determine whether the genes whose expression is most affected by loss of MYS-1 activity have higher levels of H4K16ac, we examined the top 15 % downregulated X-linked genes in *mys*-*1(RNAi)* XO hermaphrodites and X-linked genes that were not differentially expressed. We found that the levels of H4K16ac were similar in these two groups, suggesting there is no correlation between regulation by MYS-1 and H4K16ac levels (Fig. 8d). This observation is consistent with the interpretation that MYS-1 has large effects on chromosome condensation levels but only small effects on gene expression.

## Discussion

**Fig. 9:**
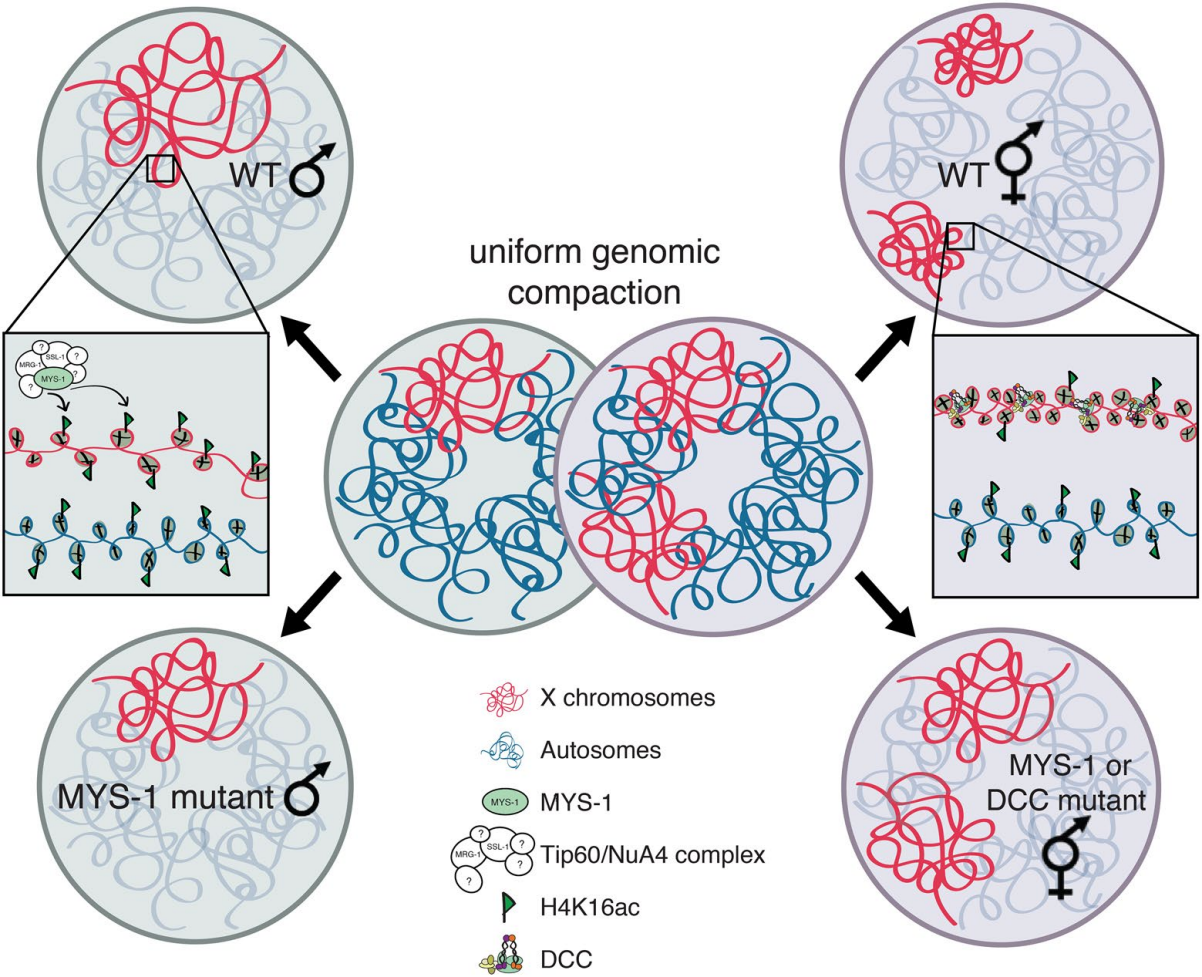
X chromosome decondensation and condensation during dosage compensation. A graphical cartoon illustrates the effects of MYS-1 and DCC on X chromosome structure in male and hermaphrodite *C. elegans*. In young embryos, the genome is uniformly compacted. The HAT MYS-1, which is a member of a putative worm Tip60-/NuA4-like complex, mediates decondensation of the X in males, while the DCC mediates condensation of the X in hermaphrodites. Additionally, MYS-1 activity is required for proper DCC localization, and therefore function, in hermaphrodites. Note that DCC-mediated compaction in hermaphrodites is accompanied by a twofold repression of X-linked gene expression, but MYS-1-mediated gene expression changes on the male X are much less significant

In this study, we sought to investigate the relationship between X chromosome-specific higher-order structural changes and gene expression levels on the highly expressed male X chromosome in *C. elegans*. According to Ohno’s hypothesis, dosage compensation in *C. elegans* is achieved in two steps: X upregulation in both sexes followed by DCC-mediated repression of the X in hermaphrodites. We established that male X chromosomes are significantly decondensed, mediated by the major H4K16 HAT, Tip60/MYS-1, suggesting the possibility that MYS-1-mediated X decondensation may be a key event in the X upregulation process. Interestingly, H4K16ac levels are nearly equivalent on the X and autosomes in XO animals. When MYS-1 is depleted in XO animals, gene expression on the X chromosome is repressed to a slightly greater degree than on autosomes. Together, our results suggest that MYS-1-mediated H4K16ac is a key factor in X decondensation, but it plays only a modest role in X upregulation in male *C. elegans*. The effects of MYS-1 and the DCC on X chromosome compaction are summarized in Fig. 9.

### MYS-1 is a MYST family histone acetyltransferase with specificity for H4K16

In most species, the major H4K16 histone acetyltransferase is MOF/KAT8/Sas2 [29–32]. However, we found that MYS-1, homolog of histone acetyltransferase Tip60, is responsible for acetylating H4K16 in *C. elegans*. Both MOF and Tip60 are MYST family histone acetyltransferases. MYST proteins are defined by their catalytic MYST domain, which contains an acetyl-coenzyme A binding domain and a C2HC-type zinc finger. Additionally, MOF and Tip60 fall under the same subfamily of MYST proteins, sharing a similar chromodomain in addition to the MYST domain [35, 38, 64]. Although structurally similar, MOF and Tip60 have different biological functions. In yeast, mice and flies, the H4K16ac mediated by MOF homologs is required for maintaining open chromatin structure [29, 31, 65, 66]. Mammalian MOF mutant embryos have cells with abnormal chromatin morphology prior to undergoing death by apoptosis [65]. In flies, MOF can reduce negative supercoiling and weaken nucleosome packing, which causes chromatin decondensation [66–68]. In conjunction with regulating chromatin structure and morphology, MOF plays a role in gene regulation. The yeast MOF homolog Sas2 mediates H4K16ac and regulates the boundary between transcriptionally active and silent telomeric chromatin [12, 69]. In *Drosophila*, MOF appears to be a global transcriptional regulator when acting in the NSL complex, which can acetylate H4K5, H4K8 and H4K16ac [70]. The MOF-NSL complex binds to a subset of active promoters and regulates housekeeping genes genome wide [71–73]. In addition to global transcription, MOF plays a specialized role in dosage compensation. MOF hyperacetylates H4K16ac on the male X in flies [26], and MOF may play a role in X upregulation in mammals as well [16, 17, 19, 20]. We show that in *C. elegans,* the H4K16 HAT plays a role in regulating X chromosome structure and expression. However, in *C. elegans*, the H4K16ac HAT appears to be Tip60, rather than MOF. *C. elegans* homologs of the MOF-MSL complex members (other than RHA-1, distantly related to MLE) are lacking or have not been uncovered. MRG-1, the chromodomain protein related to MSL3 (MSL subunit), is more closely related to MRG15 (Tip60 complex subunit) than to MSL3 [74]. Therefore, *C. elegans* may not have a MOF-MSL-like complex and instead uses a Tip60-like complex to acetylate H4K16.

In vitro Tip60 can acetylate H2A (K5), H3 (K14), H4 (K5, K8, K12 and K16), as well as histone variants and non-histone proteins [42, 75]. We found that MYS-1 is responsible for H4K16ac in vivo. Tip60 activity can also play a role in gene regulation; however, it contributes to both transcriptional activation and transcriptional repression [38]. Tip60, like MOF, binds to promoters of many genes in mammals, often with other HATs [53, 76–78], consistent with a role in regulating transcription. However, Tip60 is better known for its global role in different cellular activities. It is a key regulator in cell cycle progression and DNA damage response and has the ability to acetylate key transcription factors involved in cell growth, DNA damage and apoptosis, such as c-Myc and p53 [79, 80]. In addition to acetylating these transcription factors, Tip60 can be recruited by these transcription factors to acetylate histones [81, 82]. MYS-1, the *C. elegans* homolog of Tip60, has also been implicated in cell cycle regulation [83] and cell fate maintenance [84]. We find that the *C. elegans* homolog of Tip60, MYS-1, also functions in similar roles as MOF. We postulate that in *C. elegans*, the MYS-1 complex may perform the function of both the Tip60 complex and the MOF complex, as has been suggested previously in fission yeast [85].

### Chromosome decondensation, histone acetylation and gene expression

Chromatin decondensation and acetylation are thought to correlate with transcriptional activation [86]. This correlation has been observed, for example, in polytene insect chromosomes [87], at the mouse *HoxB* gene cluster [88] and also at the β-globin locus in mice, humans and chickens [89–91]. Additionally, in vitro studies have provided extensive evidence showing H4K16ac inhibits chromatin fiber compaction by weakening nucleosome–nucleosome interactions, which is thought to create a more permissive state for transcription [34, 69, 92, 93]. A more global example is fly dosage compensation, where MOF-mediated acetylation decondenses the X chromosome which hyperactivates the single male X [26, 27].

Our results indicate that although H4K16ac is uniformly distributed on the X and autosomes in *C. elegans* XO animals (Fig. 6), the loss of H4K16ac results in condensation of the X and no detectable change in autosomes (Fig. 1). Therefore, H4K16ac and chromosome decondensation correlate on the X chromosome but not on the autosomes. Similar to what we see in autosomes, a recent study in mouse ES cells found that broad domains of histone acetylation are lost during ES cell differentiation, but this loss results in no change in compaction [52]. We find increased levels of H3K14ac and H3K56ac in MYS-1 mutants compared to wild-type worms. It is possible that the lack of condensation defects on the autosomes is due to these marks compensating for the loss of H4K16ac. This suggests that H4K16ac and chromatin compaction are not always correlated.

It is unclear why the X chromosome is more sensitive to MYS-1-mediated decondensation than autosomes, despite near equal levels of H4K16ac, examined both globally and at genic regions. The X chromosome may be more sensitive to histone acetylation-mediated changes in compaction due to its unique properties. We did find that the X chromosomes contain proportionally more intergenic regions compared to autosomes, and their acetylation may contribute to decondensation of the X chromosome. In addition, MNase-seq analysis in *C. elegans* has found that on average X-linked gene promoters have higher nucleosome occupancy compared to autosomal promoters [94]. Furthermore, the autosomes have two large heterochromatic regions associated with the nuclear lamina at their ends, while the X chromosome only has one such region [95]. Due to these and other differences, the X may respond differently to changes in acetylation levels than the autosomes.

In addition, we found a greater degree of change in X chromosome compaction compared to change in X-linked gene expression in MYS-1-depleted XO worms, suggesting that chromatin decondensation does not necessarily result in increased transcription. These observations are reminiscent of phenotypes reported in flies deficient for the function of the chromatin remodeling protein ISWI. *Iswi* mutant male larvae have bloated X chromosomes, suggesting that the X is more sensitive to the loss of ISWI function than autosomes [96]. Decondensation of the X requires MOF activity [28]. However, gene expression defects in *Iswi* mutants are more global, and not X-specific, despite the X-specific defects in chromatin folding [97]. Separation of chromatin compaction and transcriptional regulation has also been seen in other contexts. For example, mutant versions of acidic activator domains can induce chromatin decondensation by recruiting HATs and chromatin-modifying proteins, but without activating transcription [98]. Chromatin decondensation in the absence of transcriptional activation is also sufficient to alter nuclear organization in mouse ES cells [99]. We also found that MYS-1 is able to mediate male X chromosome decondensation even when transcription is severely reduced (Fig. 5). These results are different from prior studies in which inhibition of transcription led to condensation of chromosome territories [49, 50]. Overall, our results suggest that increased transcription and chromatin decondensation may create a permissive chromatin state, but they are not sufficient to cause transcriptional activation. Furthermore, high levels of transcription are not necessary for large-scale changes in chromatin compaction.

### X decondensation and upregulation

Ohno hypothesized that X chromosome gene expression between the sexes must be balanced, as well as X to autosomal expression within a sex [1]. Whether X upregulation, the mechanism that balances the X to autosomal gene expression in males, occurs in *C. elegans* is still being debated. Current evidence [15, 19, 21, 23], including our gene expression analysis (Fig. 7a), suggests that in the absence of DCC-mediated repression in males or in DCC mutant hermaphrodites, on average X-linked genes are more highly expressed per copy than autosomal genes. However, if the male X was indeed upregulated over evolutionary time, the mechanism is more likely to be gene by gene rather than chromosome wide [24].

Our study adds to the body of evidence concerning the regulation of the X chromosome. Our data show that the *C. elegans* H4K16 HAT, MYS-1, is decondensing the male X chromosome (Fig. 9), similar to what is observed on the fly male X [66]. *Mof* mutant mice [65] and ES cells [100] also exhibit abnormal chromatin condensation, although more globally. However, in all cases, including our data, it is unclear whether the condensation defects are due to the lack of H4K16ac or some other activity attributable to the HAT. Ohno's hypothesis would suggest that in hermaphrodites, MYS-1-mediated decondensation is counteracted by DCC-mediated compaction. However, MYS-1 also affects DCC localization. Therefore, in *mys*-*1* mutant hermaphrodites, both MYS-1 and DCC functions are compromised, making it difficult to dissect individual contributions to chromosome condensation phenotypes (Fig. 9). While DCC-mediated compaction in hermaphrodites is accompanied by twofold gene repression, the effects on X-linked gene expression in MYS-1-depleted males are minor (Fig. 7). This result is very different from flies, where a mutation in *mof* causes a twofold effect on gene expression on the X chromosome [26], but is comparable to the magnitude of the effect of Mof depletion in mouse ES cell X chromosomes [101] or on autosomes of male flies [57]. Minimal X bias in gene expression changes after *mys*-*1* depletion is also consistent with the observation that reduced MYS-1 activity has similar effects on male and hermaphrodite viability (Fig. 2f). Gene expression levels change significantly for a few X-linked genes after MYS-1 depletion, and it is possible that for these genes, MYS-1-mediated regulation is a significant component of the upregulation mechanism. Thus, MYS-1 may act as one of several gene-by-gene X-upregulatory mechanisms, acting on a subset of genes. Alternatively, the bias toward the X chromosome for MYS-1-mediated regulation may reflect the unique gene content of the chromosome rather than an X-upregulatory process.

## Conclusions

Although the hypothesis of X upregulation has been around for several decades, it was not until recently that evidence began to emerge showing that the X chromosomes are in fact highly expressed in mammals and worms. Our gene expression analysis is consistent with the hypothesis that X upregulation occurs in *C. elegans* males, as we see an X:A expression ratio much higher than 0.5. We show that a TIP60-like complex acetylates H4K16 and decondenses the X in *C. elegans* males, and this decondensation may contribute to the upregulation of expression of some genes on the chromosome. H4K16ac is a key factor in male-specific X upregulation in *Drosophila* [26, 27] and has been implicated in the same process in mammals [101]. However, MYS-1 activity alone does not account for the high level of X-linked gene expression in *C. elegans*. Future studies will examine additional mechanisms that may directly contribute to regulation of X-linked gene expression.

## Methods

### Strains

All strains were maintained on NGM agar plates with *E. coli* (OP50) as a food source, using standard methods [102]. Strain includes: N2 Bristol strain (wild type); TY4403 *him*-*8(e1489)* IV; MT13172 *mys*-*1(n4075)* V/nT1 [qIs51]; MT12963 *ssl*-*1(n4077)* III/eT1; VC1931 mys-2(ok2429)/hln1 [unc-101(sy241)] I; TY1072 *her*-*1(e1520)* V; *sdc*-*2(y74)* X; and EKM71 *dpy*-*21(e428)*.

### RNA interference

*Escherichia coli* HT115 bacteria expressing double-stranded RNA for *mys*-*1, mys-2, mys*-*4, cbp*-*1, mrg*-*1*, *ssl*-*1*, *rha*-*1*, *wdr*-*5.1*, *c16A11.4*, *ama*-*1* or vector control (polylinker), were used for feeding RNAi using the Ahringer laboratory RNAi feeding library [103]. To obtain AMA-1-depleted adults, one-generation feeding RNAi was performed as follows: L1-stage *him*-*8(e1489)* larvae were placed on plates seeded with *ama*-*1* or control RNAi bacteria and were grown to adulthood and examined. To obtain AMA-1-depleted embryos, *her*-*1(e1520)* V; *sdc*-*2(y74)* X L3 stage larvae were placed on plates seeded with *ama*-*1* or control RNAi bacteria and grown to adulthood. The progeny (F_1_ generation) embryos were examined. Two-generation feeding RNAi (for all other analyses) was performed as follows; P_0_ adults from one-generation feeding RNAi were transferred to new RNAi plates to produce progeny for 24 h. These progeny (F_1_ generation) were grown to adulthood and examined.

### Fluorescence in situ hybridization (FISH)

To generate FISH probe templates, purified yeast artificial chromosome (YAC) DNA was amplified by degenerate oligonucleotide-primed PCR [104, 105]. The labeled chromosome-paint probes were prepared, and FISH was performed as previously described [10, 105].

### Microscopy and image analysis

Details of image analysis were previously described [10]. To summarize, masks (intensity threshold value) were applied over an image to distinguish real signal from background for all nuclei for each channel. DAPI mask was used as the total volume of the nucleus. The percent nuclear volume was obtained by dividing the volume of the specific chromosome over the volume of the whole nucleus. The percentages were averaged over all nuclei within an experimental set to calculate the final mean percentages. Descriptive statistics (standard deviation and sample size) were also calculated. Sample sizes are listed in each figure. Error bars shown are means ±1 standard deviation of the mean. Percent volume differences were evaluated by unpaired (two sample) Student’s t-test.

Distance measurements between two probes were described previously [10]. In brief the distance between the two probes (containing DNA amplified from YACs: either Y44D2 or Y108G6) was the distance between two separate spots closest to one another. The final median and interquartile range of the data is shown in boxplots. Sample sizes are listed in each figure. Whiskers shown indicate distribution from minimum to maximum. Probe distance differences were evaluated by unpaired (two sample) Student’s t-test.

### Immunofluorescence (IF)

Immunofluorescence experiments were performed as described [106]. Young adult worms were dissected in 1X sperm salts (50 mM pipes pH 7, 25 mM KCl, 1 mM MgSO_4_, 45 mM NaCl and 2 mM CaCl_2_, supplemented with 1 mM levamisole), fixed in 4 % paraformaldehyde in 1X sperm salts for 5 min and frozen on dry-ice for 10 min. Slides were washed three times in PBST before incubation with diluted primary antibodies in a humid chamber, overnight at room temperature. Slides were then washed three times for 10 min with PBST, incubated for 1 h with diluted secondary antibody at 37 °C, washed twice for 10 min with PBST and once for 10 min with PBST plus DAPI. Slides were mounted with Vectashield (Vector Labs). Antibodies were used at the following concentrations: H4K16ac (Millipore 07-329) at 1:500, H2A5ac (Abcam ab1764) at 1:100, rabbit anti-DPY-27 [106] at 1:100.

### Combined FISH and IF

Dissected worms were processed for FISH, as above. After the last FISH wash, slides were washed in PBST once, and the IF protocol (above) was performed beginning with the addition of primary antibody.

### Western blot analysis

Adult mutant or RNAi-treated worms were collected. Equal volume of sample buffer (0.1 M Tris pH 6.8, 7.5 M urea, 2 % SDS, 100 mM β-ME, 0.05 % bromophenol blue) was added to worms. Lysates were prepared by heating worms to 65 °C for 10 min, sonicating for two 30-s bursts, heating to 65 °C for 5 min, heating to 95 °C for 5 min and then kept at 37 °C until loading onto SDS-PAGE gel. Proteins were transferred to nitrocellulose and blotted with the following antibodies: H4K16ac (Millipore 07-329) at 1:250, H3K9ac (Abcam ab4441) at 1:1000, H3K14ac (Abcam ab5946) at 1:500, H3K56ac (Millipore 07-677) at 1:4000, H4K5ac (Upstate 07-327) at 1:500, H4K8ac (Abcam ab5823) at 1:2000, β-tubulin (Novus NB600-936) at 1:1000. Anti-MYS-1 antibodies were raised in rabbit against the N-terminal 28 amino acids (TEPKKEIIEDENHGISKKIPTDPRQYEK) and were used at 1:500.

### Worm growth and collection

Strains were maintained at 20 °C on NGM agar plates. Worm populations were synchronized by bleaching gravid adults and allowing the embryos to hatch overnight. Larval (L1s) worms were plated and grown for 24 h at 20 °C on NGM plates for L3 collection. For MYS-1 depletion, synchronized L1s were placed on seeded RNAi plates and grown to gravid adults. Embryos were collected by bleaching and hatched overnight. Larval (L1s) worms were plated and grown for 24 h at 20 °C on RNAi plates for L3 collection.

### Chromatin immunoprecipitation (ChIP)

ChIP preparation is a variation of two previous described methods [107, 108]. Frozen synchronized L3 worm pellets were crushed (pulverized by grinding in liquid nitrogen with a mortar and pestle) and cross-linked in ten volumes of 1.1 % formaldehyde in PBS plus protease and phosphatase inhibitors. The lysate was rotated at room temperature for 20 min. 2.5 M glycine was added to a final concentration of 125 mM and rocked gently for 5 min at room temperature to quench formaldehyde. Lysate was centrifuged at 4000×*g* for 3 min, and the supernatant was removed. The pellet was resuspended in 10 ml of cold PBS plus protease and phosphatase inhibitors and pelleted by spinning at 4000×*g* for 3 min. The pellet was resuspended in one volume of FA buffer (50 mM HEPES/KOH pH 7.5, 1 mM EDTA, 1 % Triton X-100, 0.1 % sodium deoxycholate; 150 mM NaCl) plus protease and phosphatase inhibitors. To obtain chromatin fragments, the lysate was sonicated on high for cycles of 30-s on and 30-s off, twice for 10 min, spun at 13,000×*g* for 15 min at 4 °C, and supernatant was collected. One milliliter of FA buffer plus protease and phosphatase inhibitors was added to the pellet and was sonicated for 15 min, spun at 13,000×*g* for 15 min at 4 °C, and supernatant was collected and combined with first supernatant. Fifty microliters of the lysate was used to make ChIP input library. Three micrograms of anti-H4K16ac (Millipore 07-329) was added to 400 μl of lysate (containing approximately 50 μl DNA). The ChIP mix was rotated overnight at 4 °C. Fifty microliters of protein G Dynabeads (Invitrogen) was added and rotated for 2 h. The beads were washed two times in FA buffer for 5 min and then washed for 5 min in FA-1M NaCl buffer (50 mM HEPES/KOH pH 7.5, 1 mM EDTA, 1 % Triton X-100, 0.1 % sodium deoxycholate; 1 M NaCl) and 10 min in FA-500 mM NaCl buffer (50 mM HEPES/KOH pH 7.5, 1 mM EDTA, 1 % Triton X-100, 0.1 % sodium deoxycholate; 500 mM NaCl). The beads were then washed for 10 min in TEL buffer (0.25 M LiCl, 1 % NP-40, 1 % sodium deoxycholate, 1 mM EDTA, 10 mM Tris–HCl pH 8.0) and then two times in TE (10 mM Tris–HCl pH 8.0, 1 mM EDTA) for 5 min. To elute the immunoprecipitation product and reverse cross-link, beads were incubated with 400 ul of ChIP elution buffer (1 % SDS, 250 mM NaCl, 10 mM Tris pH 8.0, 1 mM EDTA) at 65 °C for 2 h with agitation every 30 min (IP input lysate was treated similarly to reverse cross-link). DNA was purified using Qiagen PCR purification kit.

### ChIP-seq

A summary of all ChIP-seq data sets and replicates is provided in Additional file 6: Table S1. Raw data files, wiggle tracks of ChIP enrichment per base pair and peak bed files are provided at Gene Expression Omnibus database (http://www.ncbi.nlm.nih.gov/geo/) under accession number [GSE84307].

ChIP DNA were ligated to Illumina adaptors and amplified by PCR. Library DNA was between 200 and 550 bp in size. Single-end 50-bp sequencing was performed using Illumina HiSeq 2500 high output. Reads were trimmed for quality using the Trim Galore! v0.3.7 program from Babraham Bioinformatics (http://www.bioinformatics.babraham.ac.uk/projects/trim_galore/) and aligned to the *C. elegans* genome version WS235 with Bowtie 1.1.1 [109]. We allowed up to two mismatches, returned only the best alignment and restricted a read to map to at most four locations in the genome. MACS2 version 2.1.0 [110] was first used to filter duplicate reads and to correct for bias downsampling that was performed in either the input or the ChIP. Multiple replicates (input and ChIP) were input into MACS2 callpeak with options –broad and *P* value (−*P* 1E − 5) for peak calling and genome-wide coverage. MACS2 coverage reads were normalized to the genome-wide median coverage, excluding the mitochondrial chromosome, and final ChIP enrichment scores per base were obtained by subtracting the input coverage. For XO hermaphrodites, X and autosomes were normalized separately, due to having half the amount of X reads compared to autosomes. MACS2 coverage reads on the autosomes were normalized to the genome-wide median coverage, whereas MACS2 coverage reads on X chromosome were normalized to half the genome-wide median coverage and final ChIP enrichment scores were combined after normalization. Lastly, data sets were standardized by *z* score transformation of the ChIP enrichment values based on the presumed background. Annotation of ChIP binding sites was done using the *cis*-regulatory element annotation system (CEAS) [111] with default settings. The ChIP-seq data were visualized by IGV browser [112].

### mRNA-seq

A summary of all mRNA-seq data sets and replicates is provided in Additional file 7: Table S2, along with RPKM and differential expression analysis values. Raw data files, RNA-seq RPKM values and differential expression analysis values are provided at Gene Expression Omnibus database (http://www.ncbi.nlm.nih.gov/geo/) under accession number [GSE84307].

For RNA preparation, ten volumes of TRIzol were added to frozen synchronized L3 worms. Samples were vortexed 30″/ice 30″ for 5 min total. RNA was extracted and precipitated using the manufacturer’s instructions. The Qiagen RNeasy kit was used to clean total RNA. The TruSeq RNA Library Preparation Kit was used to prepare non-stranded mRNA-seq libraries. Single-end 50-bp sequencing was performed using Illumina HiSeq 2000. Reads were trimmed for quality using Trim Galore! and aligned to the *C. elegans* genome version WS235 with TopHat v2.0.13 [113] using default parameters, allowing up to 20 hits for each read. Gene expression was quantified using Cufflinks v2.2.1 [114] with use of “rescue method” for multireads and supplying gene annotation for WS235. Gene count estimation was performed using HTSeq-count tool v0.6.0 in the default “union” mode [115]. Differential expression analysis was performed using DESeq2 v1.6.3 [116] in R version 3.2.3. All analyses were performed with genes that had an average expression level above 1 RPKM (reads per kilobase per million, as calculated by Cufflinks).

## Additional files

**Additional file 1: Fig. S1.** Tip60-/NuA4-like complex members mediate X chromosome decondensation. (A) Representative images of X paint FISH stained nuclei of wild-type males, and *ssl*-*1(n4077)* males, and (B) males depleted of HATs and Tip60/NuA4, MOF-MSL, MOF-NSL complex members. (C) Representative images of chromosome I paint FISH of wild-type and *ssl*-*1(n4077)* males and (D) males depleted of HATs and Tip60/NuA4, MOF-MSL, MOF-NSL complex members. Scale bars equal 5 µm.

**Additional file 2: Fig. S2.** (A) H4K16ac immunofluorescence and western blot analysis of adult *mys*-*1* mutant and RNAi-treated worms. H4K16ac levels are depleted in *mys*-*1(RNAi)* and *mys*-*1(n4075)* mutant males compared to control males, both by immunofluorescence and by western blot analysis. Scale bars equal 5 µm. Tubulin is shown as a loading control. (B) Immunofluorescence and western blot analysis of adult MYS-1 mutant and RNAi-treated worms probed for additional histone acetylation marks. H2AK5ac, H3K9ac, H3K14ac, H3K56ac, H4K5ac and H4K8ac are not depleted in *mys*-*1(n4075)* mutants or in *mys*-*1(RNAi)* worms. Scale bars equal 5 µm. Tubulin is shown as a loading control. (C) H4K16ac immunofluorescence of adult MYS-2 mutants. H4K16ac levels are not depleted in *mys*-*2(ok2429)* mutants compared to wild type.

**Additional file 3: Fig. S3.** MYS-1-mediated X chromosome decondensation is evident in XO hermaphrodites. (A) Quantification of the percentage of nuclear volume occupied by X in adult XO hermaphrodites; *her*-*1(e1520)* V; *sdc*-*2(y74)* X (n = 20) and *her*-*1(e1520)* V; *sdc*-*2(y74)* X fed *mys*-*1(RNAi)* (n = 20). Error bars indicate standard deviation. Asterisks indicate level of statistical significance by t-test analysis (****P* < .001). (B) Quantification of the percentage of nuclear volume occupied by chromosome I in XO hermaphrodites; *her*-*1(e1520)* V; *sdc*-*2(y74)* X (n = 20), *her*-*1(e1520)* V and *sdc*-*2(y74)* X fed *mys*-*1(RNAi)* (n = 16). Error bars indicate standard deviation. No statistically significant differences were found (p > 0.5).

**Additional file 4: Fig. S4.** Profile of H4K16ac at *rex* sites in XX and XO animals. Representative IGV genome browser views of ChIP-seq scores for H4K16ac at *rex* sites at TAD boundaries that have the strongest *rex*–*rex* interactions in XX and XO hermaphrodites.

**Additional file 5: Fig. S5.** MYS-1 is depleted for RNA-seq. Western blot analysis of the depletion in L3 worms after MYS-1 RNAi feeding for RNA-seq samples. MYS-1 was successfully depleted. Tubulin is shown as a loading control.

**Additional file 6: Table S1.** The list of ChIP-seq data sets and their GEO accession numbers.

**Additional file 7: Table S2.** The list of mRNA-seq data sets and their GEO accession numbers, average RPKM expression levels for data sets and DESeq2 analysis results for differential expression.

## Abbreviations

DCC: dosage compensation complex
HAT: histone acetyltransferase
H4K16ac: histone H4 lysine 16 acetylation
FISH: fluorescent in situ hybridization
YAC: yeast artificial chromosome
IF: immunofluorescence
MSL: male-specific lethal
NSL: non-specific lethal
TSS: transcription start site
TTS: transcription termination site
