# Supplementary figures and images for: An H4K16 histone acetyltransferase mediates decondensation of the X chromosome in C. elegans males
Source: Epigenetics Chromatin. 2016 Oct 19;9:44. doi: 10.1186/s13072-016-0097-x (PMC5070013; doi:10.1186/s13072-016-0097-x)

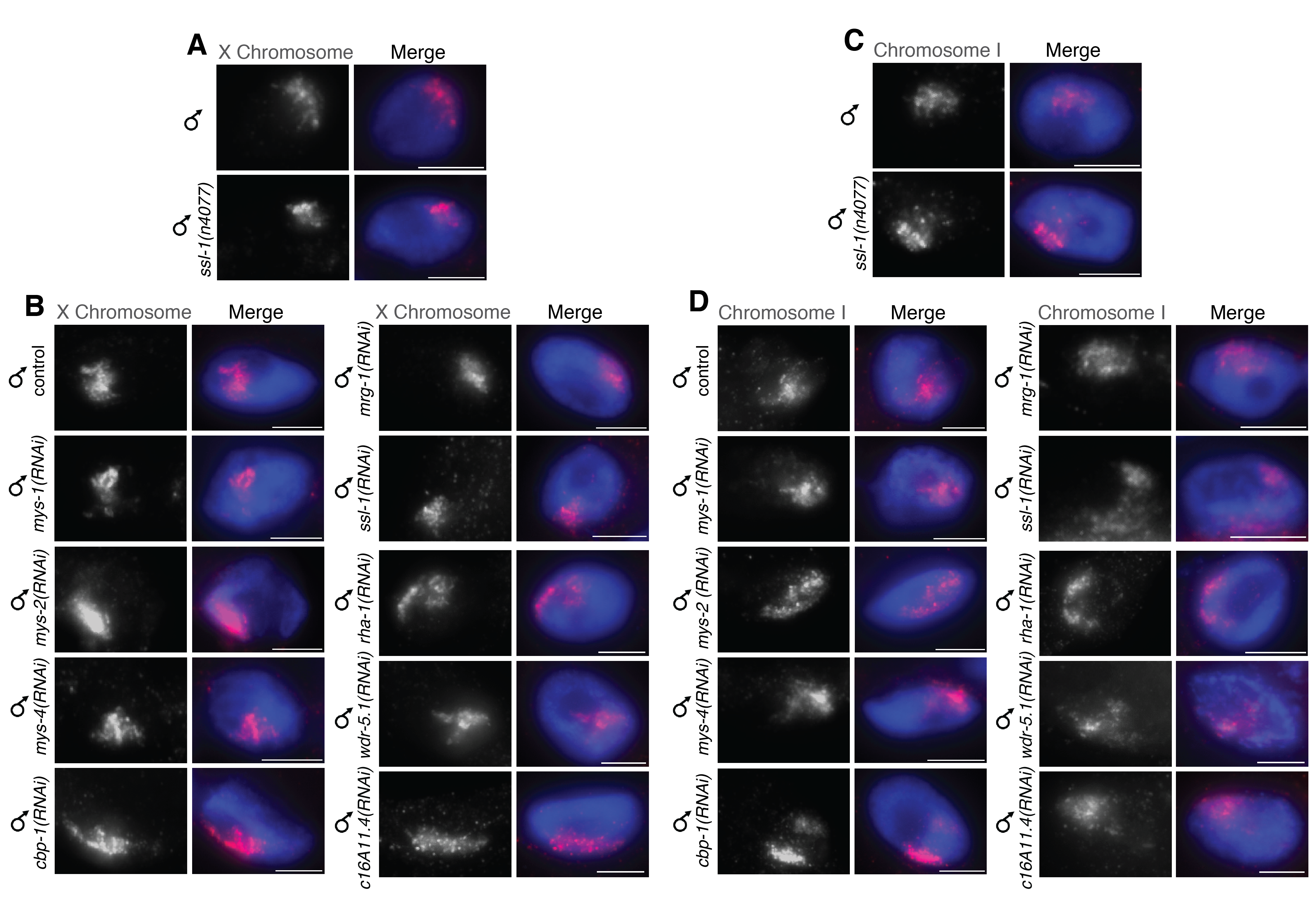

Supplement: Supplementary file 1 — Additional file 1: Fig. S1. Tip60-/NuA4-like complex members mediate X chromosome decondensation. (A) Representative images of X paint FISH stained nuclei of wild-type males, and ssl-1(n4077) males, and (B) males depleted of HATs and Tip60/NuA4, MOF-MSL, MOF-NSL complex members. (C) Representative images of chromosome I paint FISH of wild-type and ssl-1(n4077) males and (D) males depleted of HATs and Tip60/NuA4, MOF-MSL, MOF-NSL complex members. Scale bars equal 5 µm. [file 13072_2016_97_MOESM1_ESM.tif]

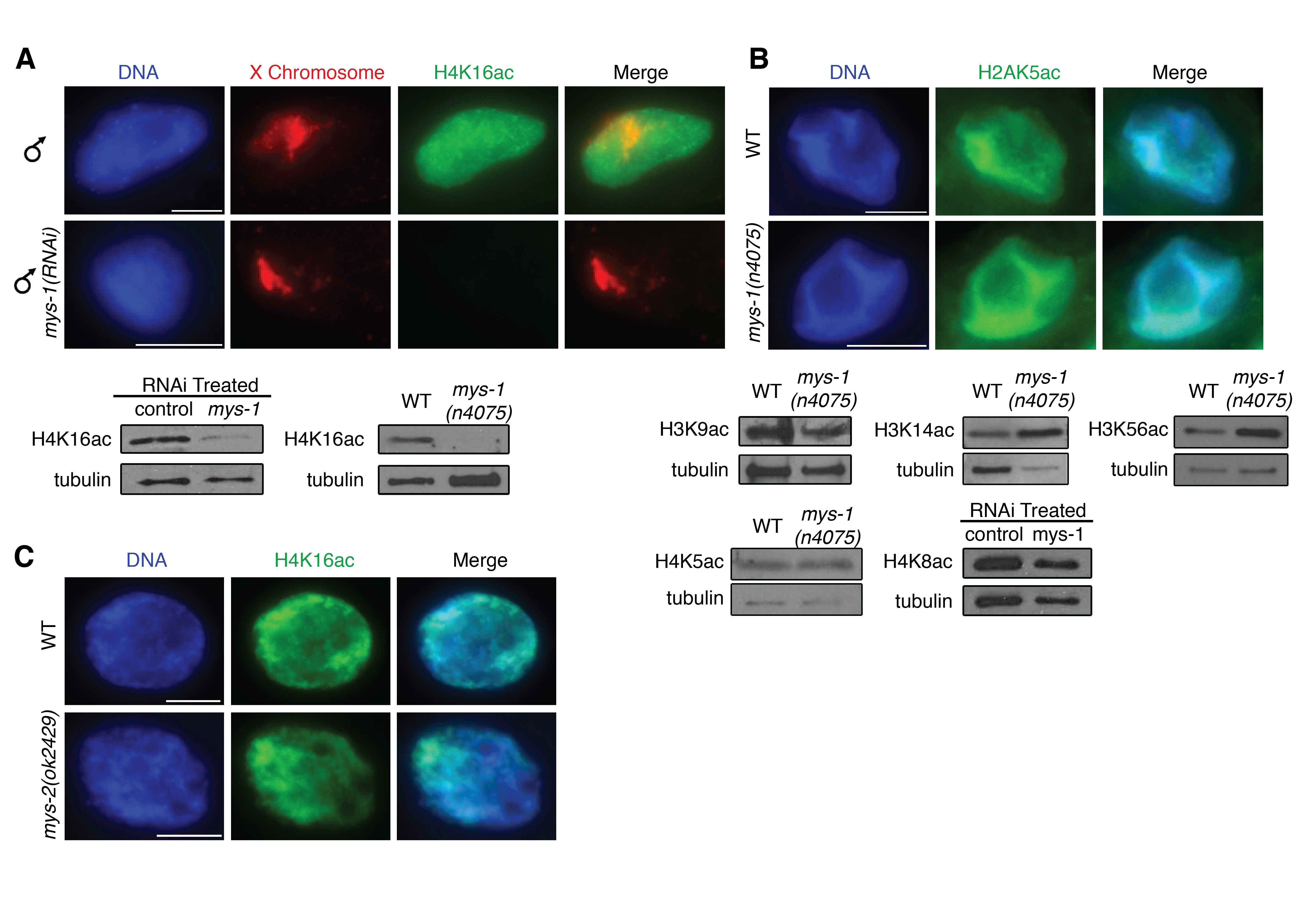

Supplement: Supplementary file 2 — Additional file 2: Fig. S2. (A) H4K16ac immunofluorescence and western blot analysis of adult mys-1 mutant and RNAi-treated worms. H4K16ac levels are depleted in mys-1(RNAi) and mys-1(n4075) mutant males compared to control males, both by immunofluorescence and by western blot analysis. Scale bars equal 5 µm. Tubulin is shown as a loading control. (B) Immunofluorescence and western blot analysis of adult MYS-1 mutant and RNAi-treated worms probed for additional histone acetylation marks. H2AK5ac, H3K9ac, H3K14ac, H3K56ac, H4K5ac and H4K8ac are not depleted in mys-1(n4075) mutants or in mys-1(RNAi) worms. Scale bars equal 5 µm. Tubulin is shown as a loading control. (C) H4K16ac immunofluorescence of adult MYS-2 mutants. H4K16ac levels are not depleted in mys-2(ok2429) mutants compared to wild type. [file 13072_2016_97_MOESM2_ESM.tif]

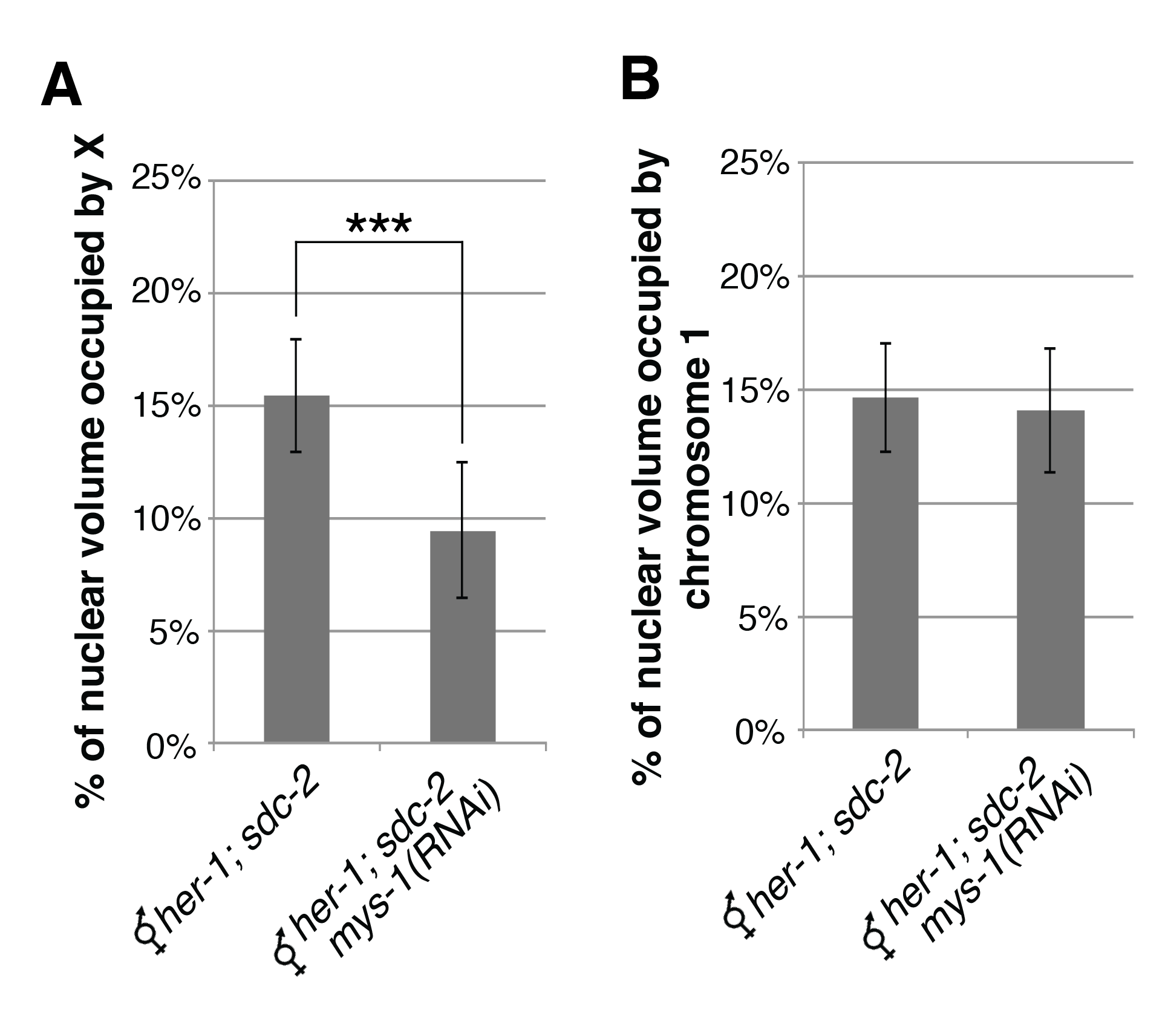

Supplement: Supplementary file 3 — Additional file 3: Fig. S3. MYS-1-mediated X chromosome decondensation is evident in XO hermaphrodites. (A) Quantification of the percentage of nuclear volume occupied by X in adult XO hermaphrodites; her-1(e1520) V; sdc-2(y74) X (n = 20) and her-1(e1520) V; sdc-2(y74) X fed mys-1(RNAi) (n = 20). Error bars indicate standard deviation. Asterisks indicate level of statistical significance by t-test analysis (***P < .001). (B) Quantification of the percentage of nuclear volume occupied by chromosome I in XO hermaphrodites; her-1(e1520) V; sdc-2(y74) X (n = 20), her-1(e1520) V and sdc-2(y74) X fed mys-1(RNAi) (n = 16). Error bars indicate standard deviation. No statistically significant differences were found (p > 0.5). [file 13072_2016_97_MOESM3_ESM.tif]

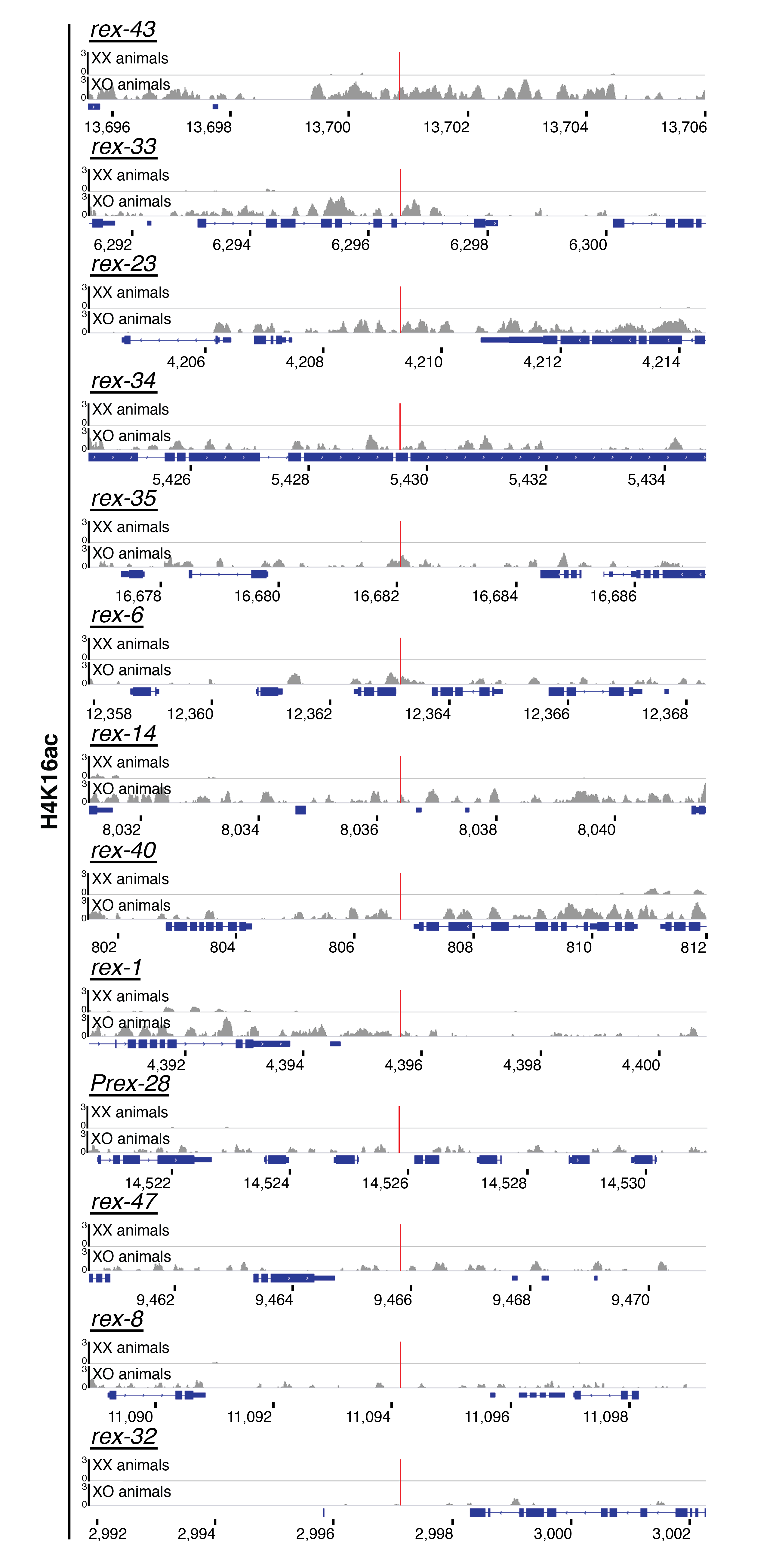

Supplement: Supplementary file 4 — Additional file 4: Fig. S4. Profile of H4K16ac at rex sites in XX and XO animals. Representative IGV genome browser views of ChIP-seq scores for H4K16ac at rex sites at TAD boundaries that have the strongest rex–rex interactions in XX and XO hermaphrodites. [file 13072_2016_97_MOESM4_ESM.tif]

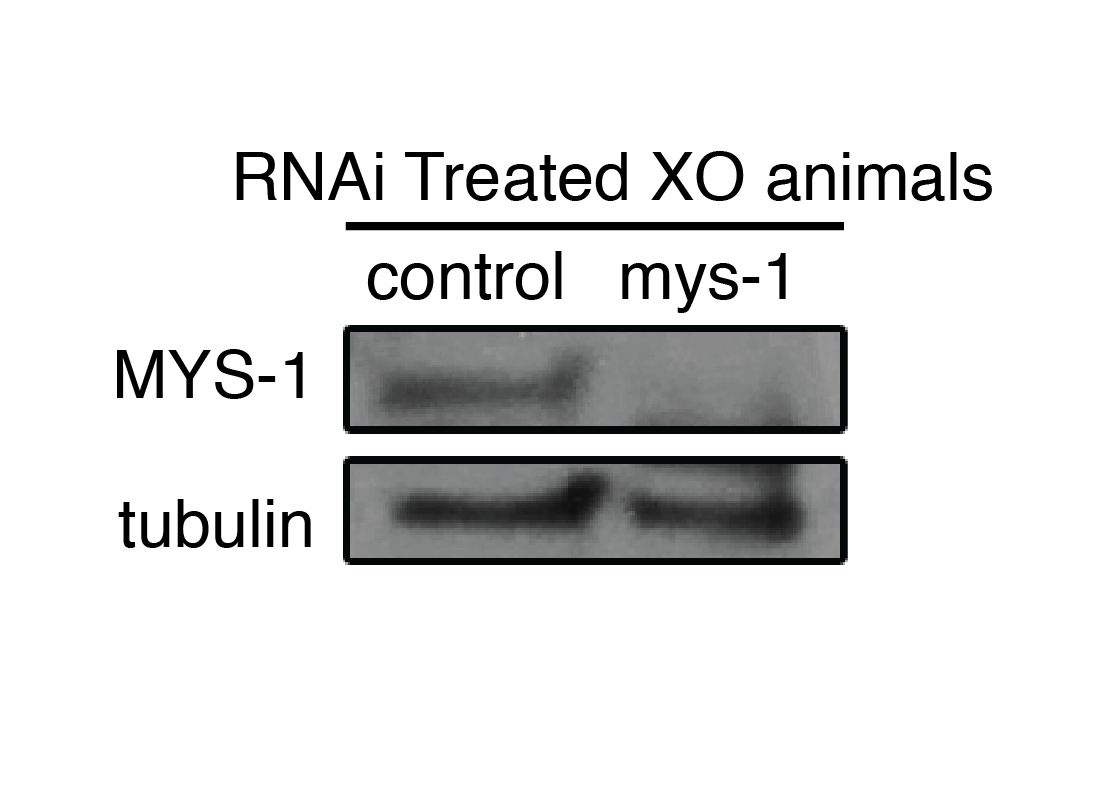

Supplement: Supplementary file 5 — Additional file 5: Fig. S5. MYS-1 is depleted for RNA-seq. Western blot analysis of the depletion in L3 worms after MYS-1 RNAi feeding for RNA-seq samples. MYS-1 was successfully depleted. Tubulin is shown as a loading control. [file 13072_2016_97_MOESM5_ESM.tif]
